# Neonatal BCG vaccination is associated with a long-term DNA methylation signature in circulating monocytes

**DOI:** 10.1126/sciadv.abn4002

**Published:** 2022-08-05

**Authors:** Samantha Bannister, Bowon Kim, Jorge Domínguez-Andrés, Gizem Kilic, Brendan R. E. Ansell, Melanie R. Neeland, Simone J. C. F. M. Moorlag, Vasiliki Matzaraki, Amanda Vlahos, Rebecca Shepherd, Susie Germano, Melanie Bahlo, Nicole L. Messina, Richard Saffery, Mihai G. Netea, Nigel Curtis, Boris Novakovic

**Affiliations:** ^1^ Infectious Diseases, Infection and Immunity Theme, Murdoch Children’s Research Institute, Parkville, VIC, Australia.; ^2^ Department of Paediatrics, The University of Melbourne, Parkville, VIC, Australia.; ^3^ Infectious Diseases Unit, The Royal Children’s Hospital, Parkville, VIC, Australia.; ^4^ Molecular Immunity, Infection and Immunity Theme, Murdoch Children’s Research Institute, Parkville, VIC, Australia.; ^5^ Department of Internal Medicine and Radboud Center for Infectious Diseases (RCI), Radboud University Nijmegen Medical Center, 6500HB Nijmegen, Netherlands.; ^6^ Population Health and Immunity Division, Walter and Eliza Hall Institute of Medical Research, Parkville, VIC, Australia.; ^7^ Department of Medical Biology, University of Melbourne, Parkville, VIC, Australia.; ^8^ Department for Immunology and Metabolism, Life and Medical Science Institute (LIMES), University of Bonn, 53115 Bonn, Germany.

## Abstract

Trained immunity describes the capacity of innate immune cells to develop heterologous memory in response to certain exogenous exposures. This phenomenon mediates, at least in part, the beneficial off-target effects of the BCG vaccine. Using an in vitro model of trained immunity, we show that BCG exposure induces a persistent change in active histone modifications, DNA methylation, transcription, and adenosine-to-inosine RNA modification in human monocytes. By profiling DNA methylation of circulating monocytes from infants in the MIS BAIR clinical trial, we identify a BCG-associated DNA methylation signature that persisted more than 12 months after neonatal BCG vaccination. Genes associated with this epigenetic signature are involved in viral response pathways, consistent with the reported off-target protection against viral infections in neonates, adults, and the elderly. Our findings indicate that the off-target effects of BCG in infants are accompanied by epigenetic remodeling of circulating monocytes that lasts more than 1 year.

## Teaser

BCG vaccine–induced DNA methylation signature can be observed in circulating monocytes more than a year after vaccination.

## INTRODUCTION

Trained immunity (TRIM) describes the capacity of innate immune cells to develop nonspecific memory characteristics in response to certain exogenous exposures through metabolic and epigenetic reprogramming (*1*). This has wide-ranging implications for immunological responses in complex diseases and infections. TRIM was originally identified in human monocytes in response to microbial exposures (*2*, *3*), but has subsequently been associated with certain metabolites and danger signals (*4*–*6*), as well as being reported in other immune (macrophages, neutrophils, natural killer cells, and dendritic cells) and even nonimmune (epithelial and endothelial) cells (*7*–*9*). The underlying mechanisms that confer TRIM and inflammatory memory occur at the level of chromatin (*10*). In monocytes and macrophages, lineage-determining transcription factors (LDTFs), such as PU.1, establish the regulatory landscape through chromatin remodeling, and the vast majority of genomic regions that are activated in response to inflammatory stimuli are marked by LDTF motifs and active posttranslational histone modifications (*11*). However, exposure to certain microbial compounds can lead to the establishment of new regulatory elements in monocytes (*12*), which influence the transcriptional response to secondary exposure (*9*, *10*). Recently, a two-step process involving cell-specific transcription factors (TFs) on the one hand [e.g., signal transducer and activator of transcription 3 (STAT3)] and general stress-responsive TFs (e.g., JUN/FOS) on the other has been described for the induction of TRIM in stem cell progenitors (*13*).

Bacille Calmette-Guérin (BCG) vaccine is one of the oldest vaccines still in routine use and remains the most commonly administered vaccine worldwide (*14*). Multiple different BCG strains exist and are in use worldwide (*15*). It is usually given at birth and is most effective in preventing childhood meningeal and miliary tuberculosis (*16*). Studies in Guinea Bissau and Uganda have shown that neonatal BCG vaccination confers protection against nontuberculous infectious diseases in early childhood (*17*, *18*). This off-target (nonspecific) protection of BCG against unrelated infections has been replicated in a controlled human infection model in which, compared with controls, BCG-vaccinated healthy adults had lower levels of yellow fever vaccine (YFV) viremia following administration of the live attenuated YFV vaccine 1 month later (*19*). Similar protection has been observed after BCG vaccination in an experimental model of human malaria infection (*20*). Last, in a clinical trial in the elderly, BCG vaccination was associated with protection against viral respiratory tract infections (*21*). These studies are the rationale for recent large-scale clinical trials of BCG vaccination in health care workers to reduce the burden of severe coronavirus disease 2019 (COVID-19) (*22*).

A large body of work has shown that the off-target effects of BCG vaccine are mediated, in part, by the induction of TRIM (*2*, *19*, *23*). Ex vivo stimulation of human monocytes 3 months after BCG vaccination with mycobacterial and nonmycobacterial stimuli results in increased production of interferon γ (IFNγ), tumor necrosis factor (TNF), and interleukin-1β (IL-1β) (*2*). This proinflammatory cytokine response is accompanied by genome-wide changes in histone modification H3K27ac and enrichment of H3K4me3 at the *TNF* and *IL-6* promoters (*2*, *19*). One year following BCG vaccination, effects on circulating monocytes remain evident, with increased surface expression of the activation markers CD11b, Toll-like receptor 4, and mannose receptor (*23*). Further studies have shown that the induction of TRIM by BCG vaccination involves long-term transcriptomic changes in hematopoietic stem cells and progenitor cells (HSPCs) that are epigenetically conveyed to peripheral monocytes at the level of chromatin accessibility (*24*, *25*).

In this study, we apply a time-course multi-omics approach (*26*) in an ex vivo TRIM model (*27*) to map the series of epigenetic and transcriptional events that take place in human monocytes during exposure to two different BCG vaccine strains. Next, we apply an epigenome-wide association study (EWAS) design to measure genome-wide DNA methylation changes in circulating (CD14^+/low^, CD16^+/−^) monocytes from 130 children (63 vaccinated with BCG and 67 matched non–BCG-vaccinated controls) from the MIS BAIR clinical trial (*28*), 14 months (range, 12 to 24 months) after vaccination. Our analysis uncovers a prominent IFN signature in genes trained by BCG in vitro and in the long-term DNA methylation signature in peripheral circulating monocytes.

## RESULTS

### Monocytes exposed to two different BCG vaccine strains display similar transcriptional and epigenetic remodeling events

**Fig. 1.**
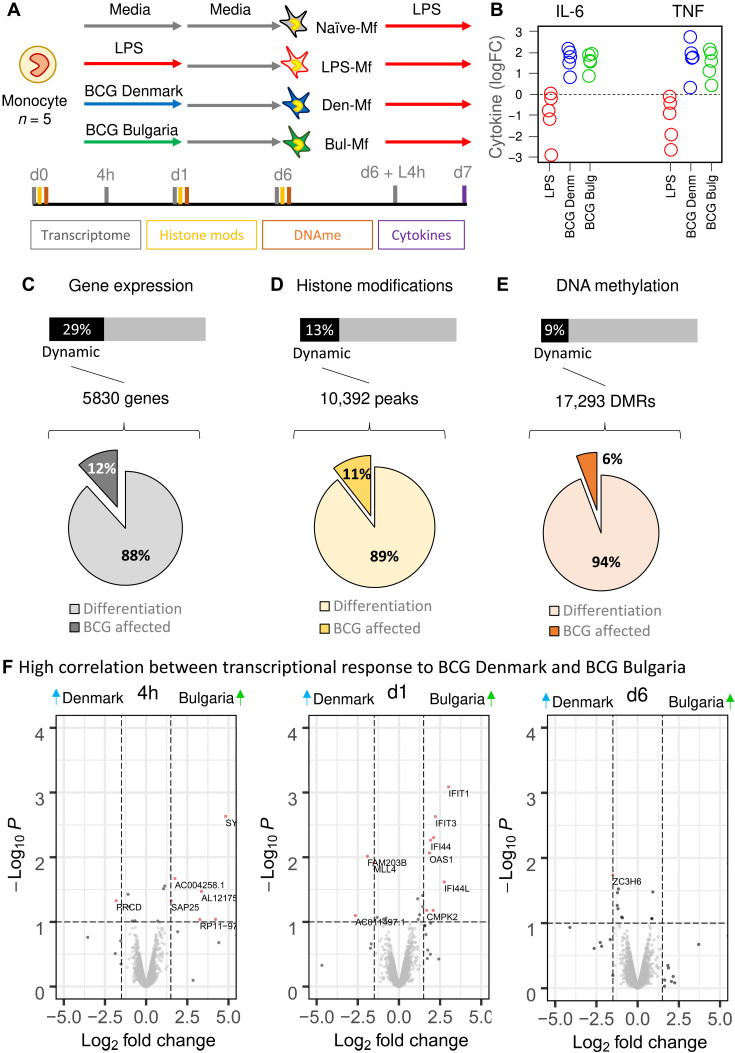
Epigenetic and transcriptional remodeling of monocytes induced by BCG vaccine in vitro. (**A**) In vitro experimental setup for epigenomic interrogation of induction of TRIM with two strains of the BCG vaccine: BCG Denmark (blue line) and BCG Bulgaria (green line) and tolerance with LPS (red line). (**B**) Macrophages derived from BCG-exposed monocytes produce higher levels of TNF and IL-6 on restimulation with LPS on day 6 relative to naïve macrophages, while those exposed to LPS show reduced levels. (**C**) A total of 5830 protein-coding genes were differentially expressed in the in vitro model, with 88% related to monocyte-to-macrophage differentiation and 12% (700 genes) affected by BCG exposure. (**D**) A total of 10,392 H3K27ac marked regions were dynamic, with 11% affected by BCG exposure. (**E**) A total of 17,293 DMRs were identified, containing 130,000 CpG sites. (**F**) Volcano plots showing differential expression between BCG Denmark– and BCG Bulgaria–exposed monocytes at 4 hours, 1 day, and 6 days after exposure. A total of 26 genes were differentially expressed across these time points, indicating a strong concordance between the transcriptional response to the two BCG vaccine strains.

Primary human monocytes (*n* = 5) were exposed to two distantly related BCG vaccine strains, BCG Denmark and BCG Bulgaria, and time-course transcriptomes, genome-wide active histone marks, and DNA methylation data were generated (Fig. 1A). At day 7, the BCG-exposed macrophages (BCG-Mf) show elevated release of TNF-α and IL-6 following a secondary lipopolysaccharide (LPS) stimulation compared to media only exposed macrophages (RPMI-Mf; Fig. 1B), in contrast to LPS-exposed macrophages (LPS-Mf) that show a tolerized phenotype (Fig. 1B), as previously described (*3*, *27*). Profiling additional cytokines linked to TRIM showed that the initial response to BCG at day 1 was most prominently marked by elevated TNF-α, granulocyte colony-stimulating factor (G-CSF), IL-2, and IL-1β, while IL-18 was elevated, but not significant (table S1). The response to restimulation was associated with elevated TNF-β, RANTES, and IL-9 (fig. S1B and table S1).

In total, 29% of protein-coding genes, 13% of H3K27ac marked chromatin, and 9% of CpG sites were dynamic during monocyte-to-macrophage differentiation or in response to BCG exposure (Fig. 1, C to E). Differentiation was the strongest driver of epigenetic change (Fig. 1, C to E), especially in the case of DNA methylation, where only 6% of differentially methylated regions (DMRs) are due to BCG exposure (Fig. 1E and fig. S1). The two BCG vaccine strains induced very similar gene expression patterns, with only 26 genes showing a significant difference in expression between monocytes exposed to the two BCG strains at any point in time (Fig. 1F and table S2). For strain-specific genes induced at 24 hours, expression in the BCG Denmark group is comparable to that of the media control, while BCG Bulgaria shows elevated level of expression. Likewise, there was a strong correlation between the two BCG strains in the cytokine release at day 7 (fig. S1B) and remodeling of transcription, H3K27ac and DNA methylation (fig. S1, C to E), indicating that monocytes respond to the two BCG vaccines in a similar manner.

### BCG induces a delayed transcriptional response in human monocytes

**Fig. 2.**
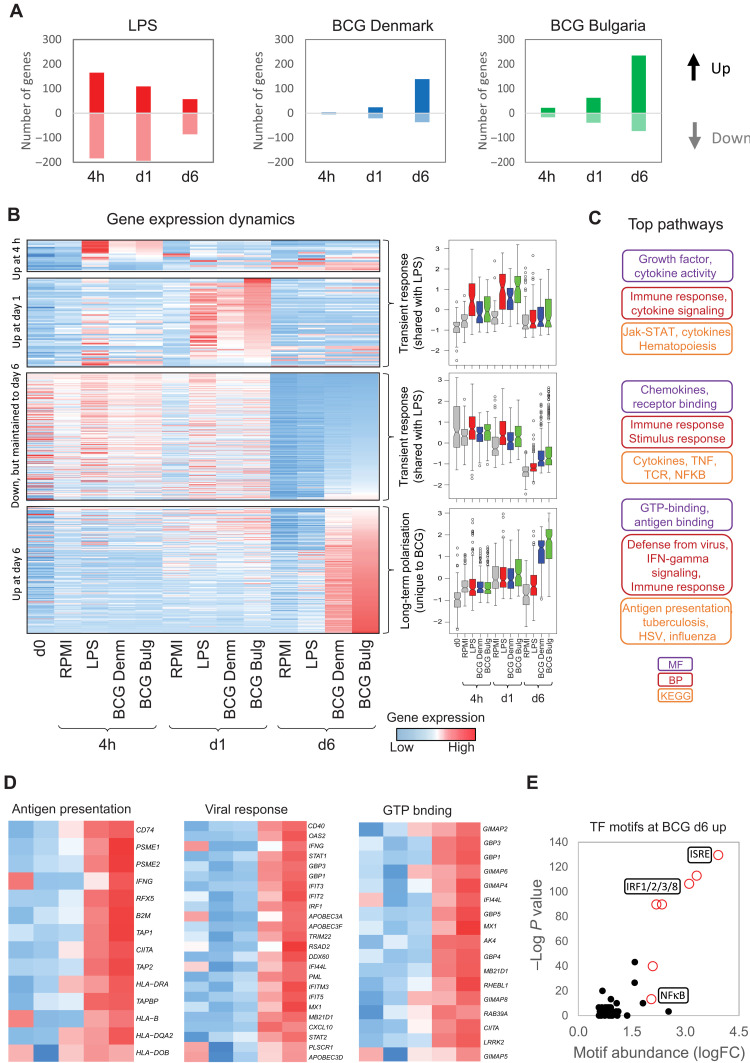
BCG exposure induces both transient and longer-term transcriptional signatures. (**A**) Bar plot showing the number of genes that are up-regulated (positive values) or down-regulated (negative values) in response to LPS or BCG Denmark or BCG Bulgaria at 4 hours, 1 day, and 6 days after exposure. (**B**) Heatmap and box plots of differentially expressed genes in response to BCG Denmark or BCG Bulgaria over time. There are two distinct sets of transcriptional dynamics: a transient up-regulation of 77 genes within the first 24 hours that is shared between BCG- and LPS-exposed monocytes, and a delayed transcriptional up-regulation of 416 genes at day 6 (5 days after stimulation) that is specific to BCG-exposed macrophages. Box plots are mean-centered RPKM values and show median, 25th, and 75th quartiles (gray, RPMI; red, LPS; blue, BCG Denmark; green, BCG Bulgaria). (**C**) Gene ontology analysis shows that the transient transcriptional response is related to cytokine release and nuclear factor κB (NFκB) signaling, while the delayed response is related to IFN signaling and response to virus. MF, molecular function; BP, biological process; KEGG, Kyoto Encyclopedia of Genes and Genomes. (**D**) Heatmap of specific gene sets that show BCG-specific up-regulation at day 6 involved in antigen presentation, viral responses [e.g., guanylate-binding proteins (GBPs)], and guanosine triphosphate (GTP) binding. (**E**) Scatter plot of −log_10_ *P* value (*y* axis) and fold change abundance (*x* axis) of a motif relative to background occurrence. Motif analysis identified the ISRE as enriched in the promoters of genes that show BCG-specific up-regulation at day 6.

The largest difference in gene expression between monocytes exposed to media only and those exposed to BCG was at day 6 (Fig. 2A and table S3). This is in contrast with the transcriptional response to LPS (Fig. 2A), and other TRIM-inducing stimuli (*26*, *29*, *30*), which is most distinct from the media control at day 1 and less pronounced by day 6 (*x* axis in fig. S2A). To illustrate this, we focused on genes associated with monocyte-to-macrophage differentiation, which are induced more quickly by some TRIM stimuli, like oxLDL, β-glucan, and heme, while LPS-induced tolerance attenuates this process (fig. S2, A and B) (*26*). BCG attenuates the expression of differentiation-associated genes similarly to LPS (fig. S2), highlighting that up-regulation of metabolic and oxidative phosphorylation–related genes is not always required for TRIM.

The initial transcriptional response to BCG at 4 hours and day 1 was transient, correlated strongly with the response to LPS (Fig. 2B), and includes cytokine signaling pathways (Fig. 2C and table S4). This response includes the up-regulation of 13 CCL and 7 CXCL chemokines (table S1), some previously implicated in TRIM, such as *CXCL1*, *CXCL2*, *CXCL3*, and *IL8* (*31*). Genes that are different between RPMI-Mf and BCG-Mf at day 6 can be separated into two categories—“lost during differentiation” and “specifically induced by BCG” (Fig. 2B). The latter group is of particular interest, because these changes in gene expression are not observed in LPS-exposed cells, nor are they present in BCG-exposed cells at 4 hours or day 1 (Fig. 2B). Therefore, this group of genes increases in expression only after BCG is removed (after 24 hours) and maintains their elevated expression 5 days later. The main pathways associated with this delayed transcriptional response are antigen presentation, complement, and viral responses (Fig. 2, C and D, and fig. S1G), while the strongest TF promoter motif signature was the IFN-stimulated response element (ISRE; Fig. 2E).

### DNA methylation signature in BCG-exposed macrophages reflects earlier histone modification and gene expression remodeling

**Fig. 3.**
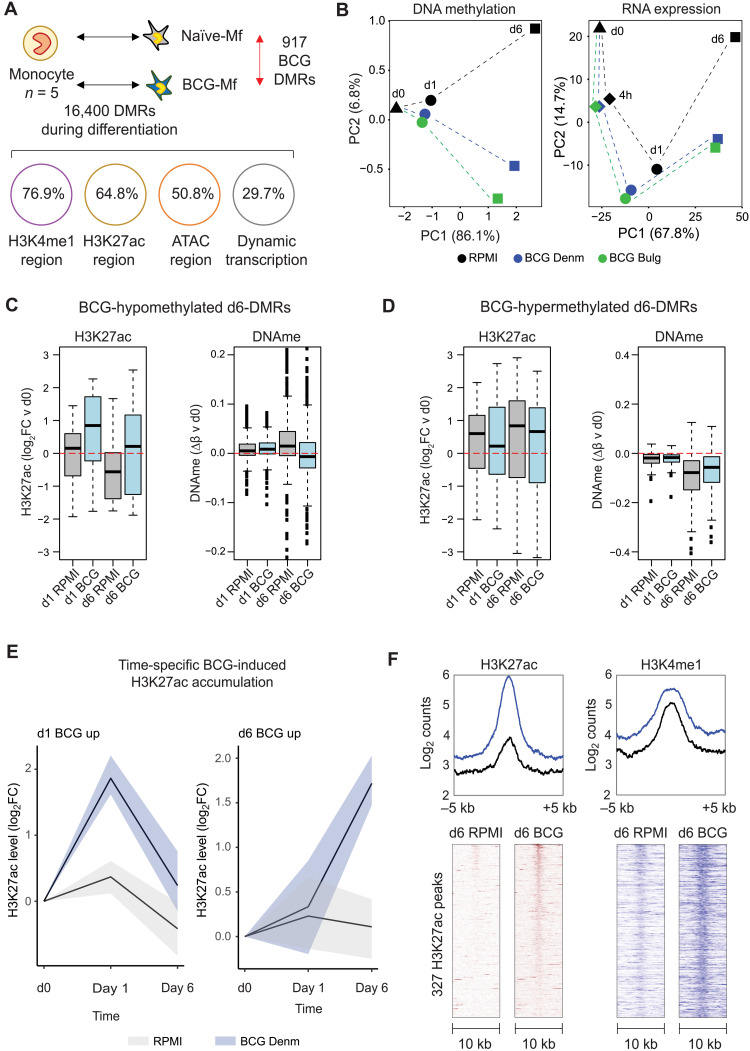
BCG-induced DNA methylation signature is preceded by posttranslational histone modification remodeling. (**A**) DNA methylation was profiled at days 0, 1, and 6 following BCG exposure, and 16,400 DMRs were related to monocyte-to-macrophage differentiation. A total of 77 and 917 DMRs were identified in both BCG Denmark– and BCG Bulgaria–exposed monocytes and macrophages at days 1 and 6, respectively. The 917 DMRs were linked to protein-coding genes, of which 29.7% were differentially expressed during differentiation and 5.6% were induced by BCG. In terms of chromatin marks, DMRs overlapped most strongly with H3K4me1 and H3K27ac regions, and to a lesser extent open chromatin [assay for transposase-accessible chromatin (ATAC) peaks]. (**B**) Principal components analysis (PCA) plot based on BCG-associated DNA methylation and transcriptional changes. In both datasets, differentiation is seen on PC1 and effect of BCG on PC2, with the BCG effect most clear at day 6. (**C** and **D**) Box plots showing change in H3K27ac signal and mean DNA methylation levels at DMRs. (C) At BCG-hypomethylated DMRs, BCG-Mfs show lower DNA methylation compared to RPMI-Mf, which is preceded by an accumulation of H3K27ac signal at day 1 in BCG-exposed macrophages. (D) At BCG-hypermethylated DMRs, the H3K27ac level in BCG-exposed cells is lower than in RPMI-exposed cells at day 1. (**E**) Line plots showing H3K27ac signal over time in RPMI-exposed (gray) and BCG Denmark–exposed (blue) monocytes (line, median; shaded area, 25th and 75th quartile). Two distinct sets of peaks are identified: those that peak at day 1 (170 peaks) and those that peak at day 6 (379 peaks). (**F**) Enriched heatmaps of BCG-Mf day 6 increased H3K27ac peaks and the corresponding H3K4me1 signal at the same peaks. Both H3K27ac and H3K4me1 show elevated levels compared to RPMI-exposed cells. Heatmaps show the center of the peak ± 5 kb.

Next, we sought to determine whether the unique transcriptional profile in BCG-Mfs was accompanied by epigenetic memory. H3K27ac is transiently dynamic during β-glucan induction of TRIM in vitro and precedes DNA methylation remodeling, which is established more slowly, but has a longer half-life (*26*). We therefore generated genome-wide DNA methylation data and H3K27ac chromatin immunoprecipitation sequencing (ChIP-seq) data at days 0, 1, and 6 and H3K4me1 data at day 6 (Fig. 1A). Several thousand DMRs were established during monocyte-to-macrophage differentiation (Fig. 3A). Like transcriptome dynamics, BCG-induced DNA methylation changes were most distinct at day 6 (Fig. 3B), with 77 DMRs at day 1 and 917 DMRs at day 6 (Fig. 3, A and B). The 917 DMRs predominantly occurred at regions marked by H3K4me1 (76%), H3K27ac (64%), and open chromatin (50%), while a third occurred near genes that show dynamic expression during differentiation (Fig. 3A). DNA methylation changes at day 6 were preceded by H3K27ac dynamics at day 1 (Fig. 3, C and D), as previously shown with β-glucan–induced TRIM in monocytes and dendritic cells responding to live virulent *Mycobacterium tuberculosis* (*32*). Next, we scanned DMRs at gene promoters and distal regions for TF motif signatures and found enrichment for ETS family members ERG, ETS1, and ETV2 at BCG distal DMRs (fig. S3A), as previously reported (*33*), highly correlating with motif enrichment at H3K27ac dynamic peaks (fig. S3B). Last, analysis of BCG-induced H3K27ac dynamics showed the same pattern as RNA expression and DNA methylation, with a distinct day 1 (170 regions) and day 6 (379 regions) signature (Fig. 3, D and E). These increases in H3K27ac signal were accompanied with increased levels of the priming histone mark H3K4me1 (Fig. 3F), as previously reported in β-glucan–induced TRIM (*26*).

### BCG-exposed macrophages are specifically trained for viral transcriptional response

**Fig. 4.**
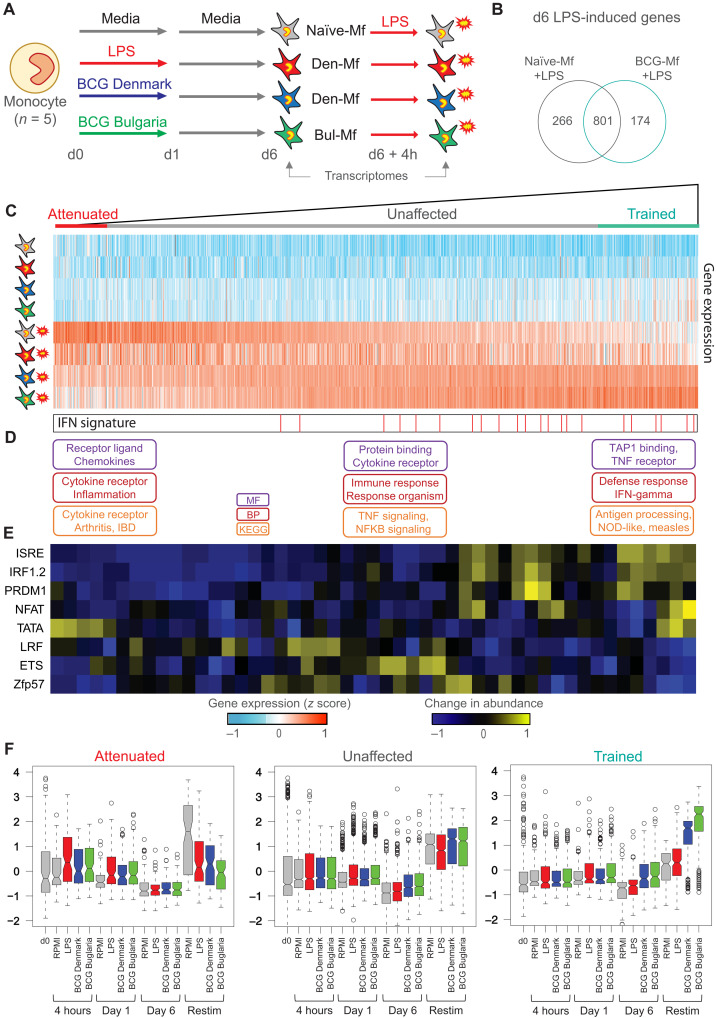
Transcriptional response to LPS reexposure in BCG-trained macrophages. (**A**) TRIM model, including data collection at 4-hour LPS reexposure at day 6. (**B**) Venn diagram showing overlap of genes induced by LPS restimulation at day 6 in Naïve-Mf and BCG-Mf. (**C**) The total macrophage transcriptional response (1242 genes) to LPS was ranked based on the induction of genes in BCG-Mfs (mean of Denmark-Mf and Bulgaria-Mf), relative to Naïve-Mfs, revealing a gradient in BCG-Mf response to LPS reexposure from trained to attenuated. Heatmap shows Naïve-Mf, LPS-Mf, and BCG-Mf at day 6 and after LPS exposure. (**D**) Gene ontology terms related to attenuated, unaffected, and trained genes, showing enrichment of IFN and viral responses on the unaffected and trained genes. (**E**) Motif enrichment analysis was performed on promoter regions using a sliding window of 60 genes from trained to attenuated. This analysis shows enrichment of IRF and PRDM1 motifs at trained genes. (**F**) Box plot showing gene expression over time for 100 attenuated, 946 unaffected, and 196 trained genes for Naïve-Mfs (gray), BCG Denmark-Mf (blue), BCG Bulgaria-Mf (green), and LPS-Mf (red). *P* values between genes in Naïve-Mf Restim group compared to other exposures within the attenuated gene set (LPS-Mf, *P* = 9.02 × 10^– 5^; Denmark-Mf, *P* = 5.86 × 10^–8^; Bulgaria-Mf, *P* = 1.56 × 10^–15^), within the equal gene set (LPS-Mf, *P* = 0.068; Denmark-Mf, *P* = 1; Bulgaria-Mf, *P* = 1), and within the trained group (LPS-Mf, *P* = 0.27; Denmark-Mf, *P* = 6.6 × 10^–42^; Bulgaria-Mf, *P* = 3.37 × 10^–61^).

To test the transcriptional response of BCG-Mf to a secondary stimulus, we exposed macrophages to LPS at day 6 (Fig. 4A). In total, 1242 genes were induced by 4-hour LPS exposure in RPMI-Mf or BCG-Mf (Fig. 4B and table S5). To identify genes that were trained or attenuated by the initial BCG exposure, we ranked genes based on their induction by the secondary LPS exposure in BCG-Mf relative to RPMI-Mf (Fig. 4C). The difference between RPMI-Mf and BCG-Mf response to LPS was a gradient, with 196 and 100 genes showing a clear trained or attenuated transcriptional response [*P* < 0.05, fold change (FC) > 2], respectively (Fig. 4C). Gene ontology analysis revealed an enrichment of genes involved in viral responses in the trained gene set (Fig. 4D and table S4), with a targetable 21-gene IFN signature (*34*, *35*) distributed across the unaffected and trained genes, but not in the attenuated gene set (Fig. 4C). Next, we scanned the promoters for motif occurrence, and due to the gradient response to LPS, we used a sliding window approach, which showed an overrepresentation of ISRE, IRF1/2, PRDM1, and NFAT at BCG-Mf–trained genes (Fig. 4E). The genes coding for the corresponding TFs were up-regulated in BCG-Mf at day 6, as was IFNγ (fig. S4C). Unlike trained genes, attenuated and unaffected gene promoters did not show a distinct motif signature (fig. S4A), with only LRF (*ZBTB7A*) enriched at attenuated genes, and Zfp57 and ETS enriched at unaffected genes (Fig. 4E).

Compared to the motif signature, remodeling of DNA methylation in BCG-Mf did not have a strong influence on trained genes, even if specific examples of coordinated H3K27ac and DNA methylation change could be observed near trained or attenuated genes (fig. S3, C to E). By plotting the expression of LPS-induced genes in macrophages, over the time course, we observe only a slight up-regulation in monocytes during the first 24 hours in response to LPS or BCG (Fig. 4F). LPS-Mf showed similar expression to BCG-Mfs at attenuated and unaffected genes at LPS restimulation, while the trained response was only observed in BCG-Mf (Fig. 4F). Trained BCG-Mf genes start to show elevated expression of genes at days 1 and 6 (Fig. 4F), which suggests that BCG-Mf are in an inflammatory trajectory preceding the LPS restimulation (fig. S4B). Of the epigenetic marks, only H3K27ac accumulation at day 6 was observed at trained genes, while attenuated and unaffected genes showed an initial peak of H3K27ac at day 1, which starts to deplete by day 6 (fig. S4D).

### IFNγ is required for BCG-induced TRIM

**Fig. 5.**
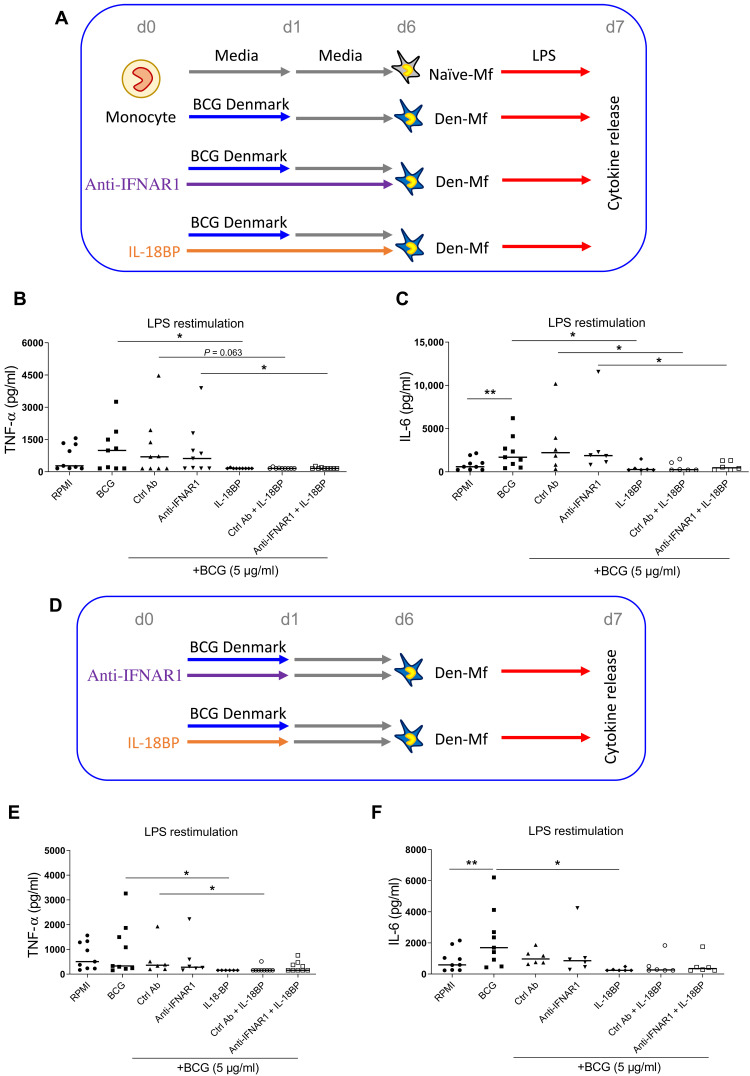
IFNγ is required for BCG-induced TRIM in vitro. (**A**) In vitro model to test the effect of IL-18BP and anti-IFNAR1 antibody (Ab) on BCG-induced TRIM. Monocytes were exposed to BCG Denmark (5 μg/ml) for 24 hours (blue line), while the anti-IFNAR1 antibody (10 μg/ml, purple line) and IL-18BP (10 μg/ml, orange line) were in the culture medium for the entire 6 days. At day 6, macrophages were exposed to LPS and cytokine release was measured 24 hours later (day 7). Dot plots show that the cytokine release in picograms per milliliter for (**B**) TNF and (**C**) IL-6 at day 7 is elevated in BCG-Mf, with the anti-IFNAR1 antibody having no effect. IL-18BP, on the other hand, completely blocks the LPS response. (**D**) In vitro model of BCG and inhibitor costimulation for 24 hours only, followed by media for 5 days, before LPS restimulation. (**E** and **F**) TNF and IL-6 production is attenuated by 24-hour IL-18BP exposure. *P < 0.05 and **P < 0.01.

Our epigenomic data strongly indicate a role for IFN signaling in BCG-induced TRIM (Figs. 2B and 4B), as shown in mouse bone marrow–derived macrophages (BMDMs) (*25*). To functionally test this, we blocked IFNγ production using IL-18BP (*36*) and type I IFN signaling using the anti-IFN α receptor 1 (IFNAR1) antibody anifrolumab, which is in clinical trials to treat systemic lupus erythematosus (*34*). There was a significant reduction in IL-6 and TNF-α production at LPS restimulation when cells were exposed to IL-18BP alone compared to cells incubated with culture medium or the anti-IFNAR1 antibody (fig. S5). Next, the inhibitors were added to the culture medium for the entire 6 days of the in vitro TRIM protocol (Fig. 5A), and TNF and IL-6 cytokine release level was used as the determinant of BCG-induced TRIM phenotype. IL-18BP blocked BCG Denmark–induced TRIM, while the anti-IFNAR1 antibody did not (Fig. 5, B and C). This suggests that IFNγ is specifically required for the elevated cytokine release observed in BCG-Mf. Because we observed distinct early (day 1) and late (day 6) BCG-induced transcriptional and H3K27ac dynamics, we next wanted to test whether blocking IFNγ only during the first 24 hours was adequate to block TRIM (Fig. 5D). This was indeed the case, with a 24-hour costimulation with BCG Denmark and IL-18BP being enough to prevent a trained phenotype at day 6 (Fig. 5E). This result indicates that IFNγ production during the first 24 hours was necessary for the subsequent trained phenotype at day 6.

### BCG vaccination is associated with a long-term DNA methylation signature in circulating monocytes

**Fig. 6.**
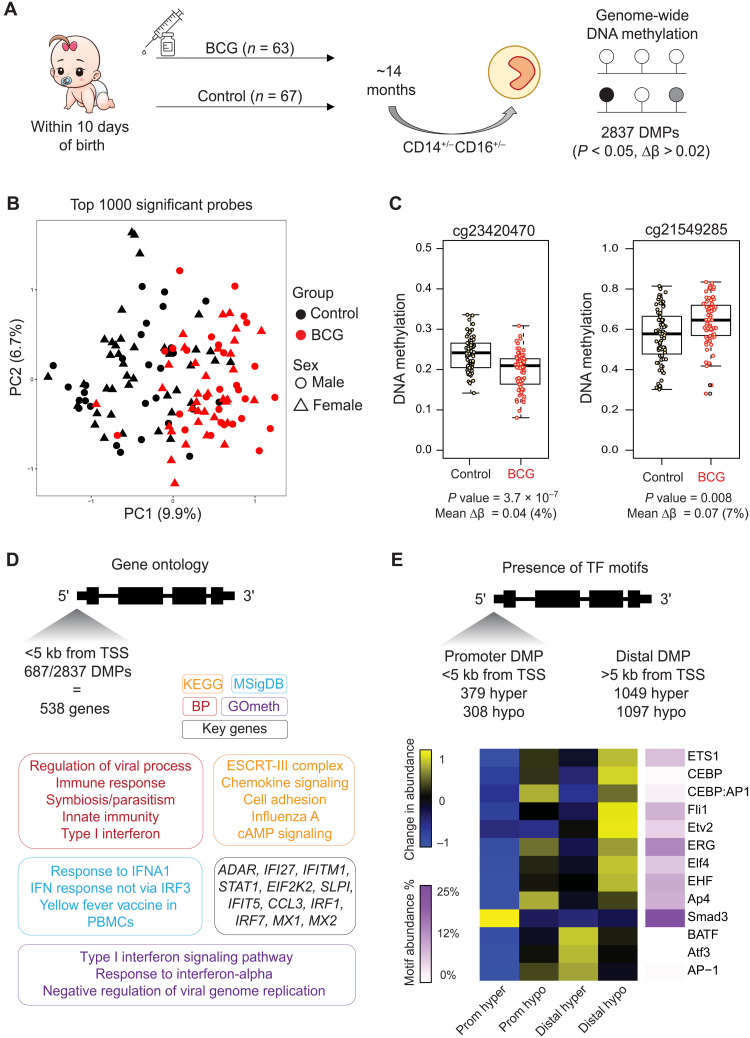
EWAS identifies BCG-associated DNA methylation signature in circulating monocytes 14 months after vaccination. (**A**) MIS BAIR clinical trial and experimental setup. Blood was collected from infants in the BCG-vaccinated group (*n* = 63) or non–BCG-vaccinated group (*n* = 67) on average 14 months after neonatal randomization (range, 12 to 24 months). Monocytes were sorted from peripheral blood mononuclear cells, and DNA methylation was quantified. A total of 2837 DMPs were identified. (**B**) PCA plot of the top 1000 DMPs shows a clear separation between the BCG-vaccinated (red) and non–BCG-vaccinated (black) groups on PC1. (**C**) Box plot and dot plot of DNA methylation for individual nonvaccinated and BCG-vaccinated samples at two probes with the lowest *P* value and highest contribution to PC1 in (B). (**D**) Gene ontology analysis was performed on 538 genes that had a promoter DMP. Gene-based (KEGG, BP, and MF) ontology analysis was performed with HOMER and showed agreement with CpG-based ontology analysis (GOmeth tool). Top genes were involved in IFN and viral responses. (**E**) Motif scanning identified distinct enrichment at promoter and distal DMPs. ETS family motifs were enriched at both promoter and distal hypomethylated DMPs, while ATF3 was enriched at distal hypermethylated DMPs.

Next, we sought to determine whether the epigenetic alterations persist for as long as the reported beneficial off-target effects of BCG vaccination in newborns (*17*). Using samples from the MIS BAIR randomized controlled trial (Table 1), we isolated total circulating monocytes (CD14^+/low^/CD16^−/+^) and performed an EWAS using 63 BCG-vaccinated and 67 matched non–BCG-vaccinated controls at 14 (range, 12 to 24) months after randomization (Fig. 6A and fig. S6). Neonates are the main recipient population of BCG vaccination worldwide and from which most epidemiological data on the heterologous effects are derived (*17*). There were no statistical differences in proportions of classical, intermediate, and nonclassical monocytes between the BCG-vaccinated and non–BCG-vaccinated groups (fig. S6, A and B).

**Table 1.** Clinical data of healthy volunteers included in the study.

**Characteristic**	**BCG-naïve, no. (%)**	**BCG-vaccinated, no. (%)**
Total	67	63
Sex (female)	28 (41.8%)	28 (44.4%)
Gestational age, weeks, mean ± SD	39.3 ± 1.2	39.3 ± 1.6
Birth weight, grams, mean ± SD	3472.3 ± 474.6	3429.0 ± 552.0
Mode of delivery (vaginal birth)	42 (62.7%)	40 (63.5%)
Maternal age at delivery, years, mean ± SD	32.9 ± 4.5	32.3 ± 4.3
Age at blood sampling, days, mean ± SD	428.4 ± 70.9	427.3 ± 57.3
Measles, mumps, and rubella (MMR) vaccination before blood sampling	53 (79.1%)	48 (76.2%)
Household smoking in first year of life	4 (6.0%)	10 (16.1%)
Ethnicity		
Caucasian	56 (83.6%)	51 (81.0%)
Asian	0	3 (4.8%)
Other	11 (16.4%)	9 (14.3%)
Maternal BCG vaccination status		
Yes	11 (16.4%)	10 (15.9%)
No	51 (76.1%)	49 (77.7%)
Not reported	5 (7.5%)	4 (6.4%)
Duration of breastfeeding		
Never	1 (1.5%)	0
<6 months	19 (28.4%)	18 (28.6%)
6–12 months	14 (20.9%)	18 (28.6%)
>12 months	33 (49.2%)	25 (39.7%)
Unknown	0	2 (3.2%)
Monocyte proportions		
Classical	86.0 ± 5.9	84.9 ± 5.1
Intermediate	5.5 ± 2.6	5.5 ± 2.7
Nonclassical	8.4 ± 4.7	9.3 ± 4.3

We identified 2836 differentially methylated probes (DMPs), with similar distribution of probes showing higher and lower DNA methylation in the BCG-vaccinated group (fig. S6, C to E, and table S6). The DNA methylation signature clearly separates the BCG-vaccinated and non–BCG-vaccinated samples (Fig. 6B), with top probes contributing to PC1 shown in Fig. 6C. An interesting observation was that BCG-associated DMPs were also present on the X chromosome, clearly separating males from females and BCG-vaccinated from non–BCG-vaccinated infants (fig. S6, F and G). Gene ontology analysis of 687 genes with a DMP in their promoter revealed enrichment for several antiviral and IFN signaling pathways, with numerous genes occurring in multiple terms including *ADAR1*, *IFITM1*, *STAT1*, *IRF1*, *IRF7*, *MX1*, and *MX2* (Fig. 6D and table S4). This enrichment was confirmed using GOmeth, a gene ontology tool that uses DNA methylation as input, with “type I IFN” and “negative regulation of viral replication” as key terms (Fig. 6D). Motif enrichment at differential probes showed similar patterns to that observed at in vitro BCG-induced DMRs, with ETS and ERG prominent at promoter and distal probes that lose DNA methylation. Distal probes that gain DNA methylation were enriched for ATF3 and BATF motifs, while the SMAD3 motif was the only one enriched at promoter probes that gain DNA methylation (Fig. 6E).

Noncoding genetic variation can influence cytokine production and induction of TRIM (*37*). To test whether our DMP-associated genes have known single-nucleotide polymorphisms (SNPs) that influence BCG training, we used a list of cis cytokine quantitative trait loci (cQTLs) from the 300BCG Project (*38*). Using 494 QTLs at 24 genes that influence BCG-trained cytokine response (BCG-cQTLs) to a microbial exposure in vitro (*Staphylococcus aureus*), we saw no direct overlap with the identified DMPs. However, 21% (5 of 24) of BCG-cQTL genes were near a DMP in our analysis, including *IL6* and *IL36B* (fig. S7 and table S7).

### BCG-associated DMRs occur near IFN-stimulated genes that are also modulated by BCG in human monocytes and mouse hematopoietic stem cells

**Fig. 7.**
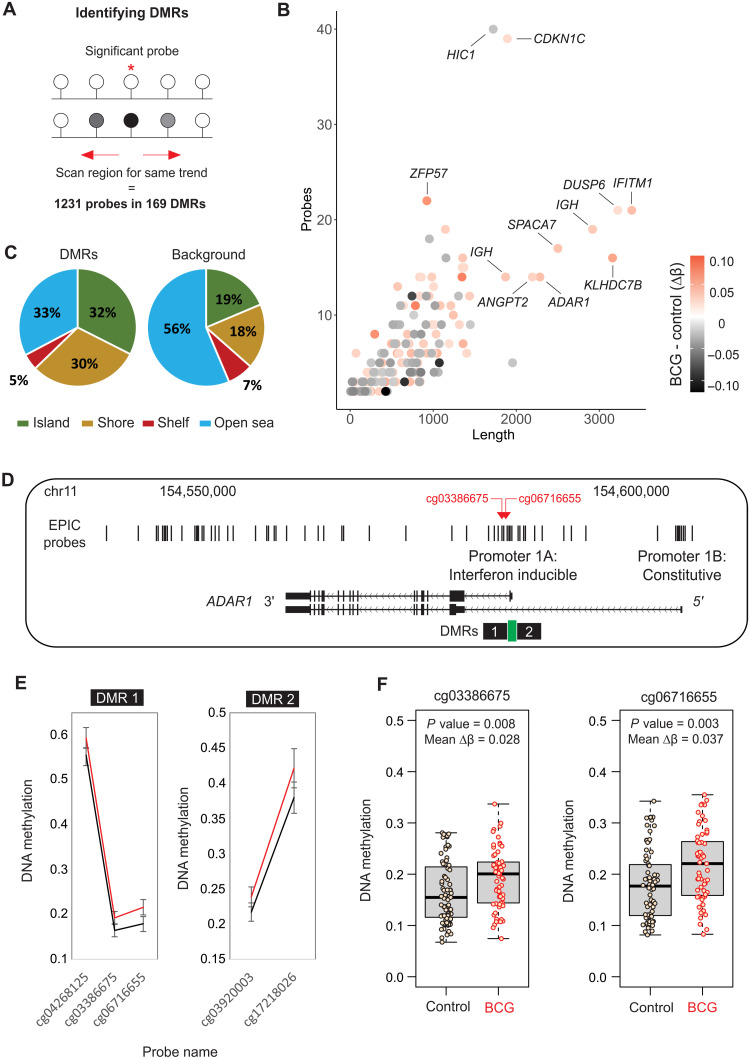
DMRs associated with BCG vaccination. (**A**) Strategy for identifying DMRs or clusters of DMPs. (**B**) Summary of the size (*x* axis) and number of probes (*y* axis) for each DMR, colored by mean change in DNA methylation between non–BCG-vaccinated and BCG-vaccinated groups. (**C**) Pie chart showing the genomic context of DMRs around CpG islands. Compared to all EPIC probes, probes within DMRs are enriched for islands and shores and depleted for open-sea regions, suggesting enrichment at regulatory regions near gene promoters. (**D**) DMR at the IFN-inducible promoter of the *ADAR1* gene. (**E**) Two DMRs occur at either side of a CpG island, with higher methylation in the BCG-vaccinated group (red line) compared to nonvaccinated group (black line). (**F**) Top probes in the DMR shown as box plot in control and BCG-vaccinated groups.

Coordinated change in DNA methylation across several CpG sites within a genomic region (DMR) is a strong indication that chromatin remodeling took place. To identify these DMRs, we scanned for DMPs that occur in clusters (Fig. 7A and table S8). The 169 DMRs ranged from 6 to 3200 base pairs (bp) in length, contained 2 to 40 CpG sites (Fig. 7B), and were more likely to occur at CpG islands and CpG shores, which are regulatory regions with high CG content usually found at gene promoters (Fig. 7C). Notable examples include *IFITM1*, which had the largest DMR (fig. S8, A and B), and *ADAR1* (fig. S8, C and D), with a DMR in its promoter (Fig. 7, D to F).

**Fig. 8.**
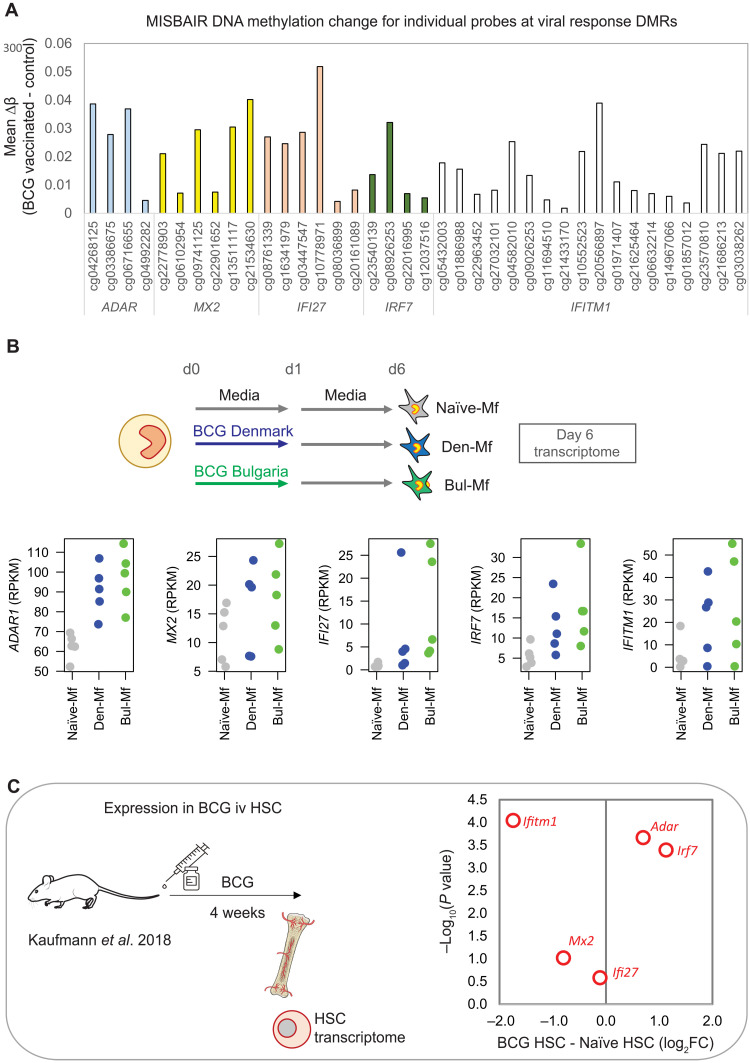
Genes with IFN-associated DMRs show altered expression in human monocytes exposed to BCG in vitro and in mouse HSCs. (**A**) Bar plot showing mean DNA methylation change between non–BCG-vaccinated and BCG-vaccinated groups for five DMRs associated with IFN type I response: *ADAR*, *MX1*, *IFI27*, *IFITM1*, and *IRF7*. (**B**) Dot plot showing gene expression of the five genes at day 6 in adult macrophages. All five genes are more highly expressed in BCG-Mf (Denmark-Mf in blue, Bulgaria-Mf in green) compared to Naïve-Mf (gray). (**C**) Expression of the five genes is altered in mouse HSCs, 4 weeks after intravenous (iv) BCG. Red dots: log_2_ fold change in BCG HSCs relative to naïve HSC.

Five genes with promoter DMRs were enriched in several antiviral pathway—*ADAR1*, *MX2*, *IFI27*, *IRF7*, and *IFITM1* (Fig. 8A). All five genes were also more highly expressed in human macrophages derived from in vitro BCG-exposed monocytes (Fig. 8B), indicating that persistent expression of these genes is a response to direct BCG exposure. However, the DNA methylation signature in circulating monocytes more than 1 year after vaccination is likely a remnant of molecular remodeling events in hematopoietic stem cells (HSCs). To explore whether these genes are modulated by BCG in HSCs, we used transcriptome data of mouse HSCs isolated 4 weeks after BCG vaccination (*25*). This analysis reveals that the expression of *Adar1* and *Irf7* is induced, while the expression of *Ifitm1* and, to a lesser extent, *Mx2* is down-regulated by BCG exposure (Fig. 8C).

### BCG-induced ADAR1 expression is associated with higher rates of adenosine-to-inosine RNA editing

**Fig. 9.**
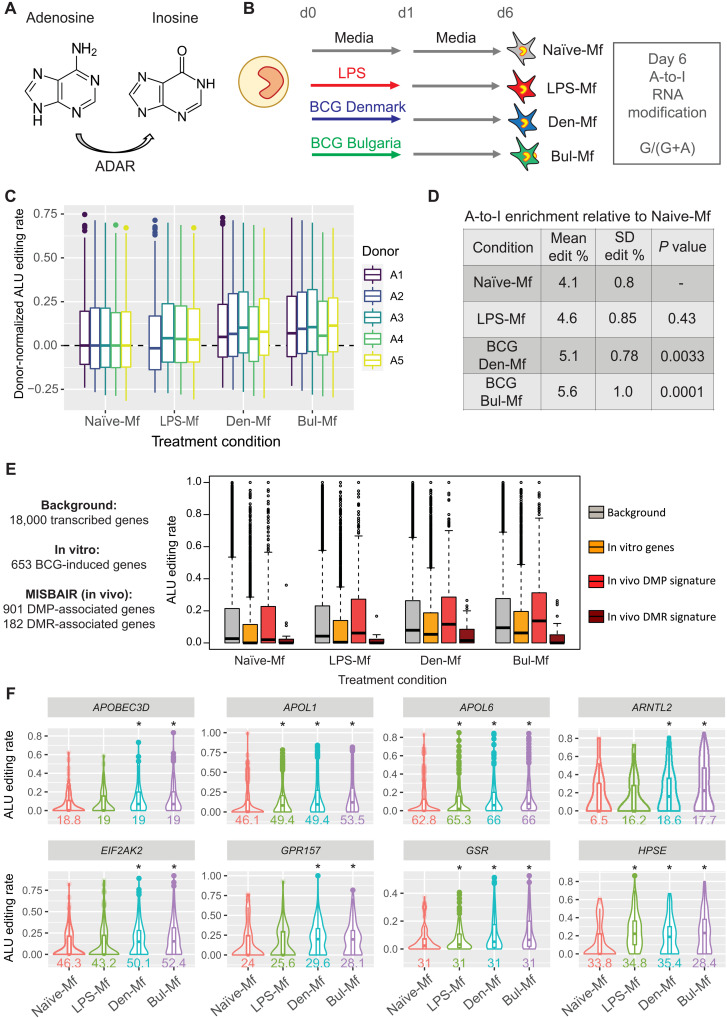
BCG induces A-to-I modification of RNA in human macrophages. (**A**) ADAR catalyzes the A-to-I modification at Alu repeats in RNA. (**B**) In vitro BCG TRIM model, showing that day 6 RNA-seq data were used to quantify A-to-I editing, read as a change from an A to a G at noncommon SNPs. (**C**) Box plot showing Alu editing rates per donor (A1 to A5) for Naïve RPMI–exposed (N), LPS-exposed (L), BCG Denmark–exposed (D), and BCG Bulgaria–exposed (B) macrophages. (**D**) A significant increase in A-to-I editing level is observed in BCG-Mf. (**E**) A-to-I editing sites were mapped to specific gene sets. Box plot shows editing proportions in a background gene set (gray), in vitro BCG-induced genes (yellow), and genes near a DMP (coral) or DMR (brown) in the MIS BAIR EWAS. In vitro induced genes and those with a DMP in vivo are particularly edited in BCG-Mf. (**F**) Violin plots showing proportion of A-to-I (A>G) at example genes that belong to the in vitro induced gene set (first row) or the in vivo DMP-associated genes (second row). Alu editing rate is shown in Naïve-Mf (red), LPS-Mf (green), Denmark-Mf (blue), and Bulgaria-Mf (purple). Conditions with significant change in editing compared to Naïve-Mf are displayed with a white background and an asterisk. The average number of edited sites detected per donor is displayed below each plot.

Last, we explored the potential consequence of higher *ADAR1* (adenosine deaminase RNA specific) expression in BCG-Mf by quantifying the level of adenosine-to-inosine (A-to-I) editing of RNA (Fig. 9A). A-to-I editing occurs at Alu repeats primarily in introns and 3′ untranslated regions and, as inosine mimics the base-pairing chemistry of guanosine, was quantified as a change from adenosine to guanine (fig. S9A) using RNA sequencing (RNA-seq) data at day 6 (Fig. 9B). We identified a significant increase in the proportion of edited sites in BCG-Mf compared to RPMI-Mf globally (Fig. 9, C and D). BCG-associated increase in A-to-I rates was not observed at day 1, suggesting that this response is not immediate but accumulates over time after BCG exposure. Endotoxin-tolerized LPS-Mf also showed a slight increase in A-to-I but did not reach significance (Fig. 9D). Next, we performed a gene set enrichment analysis to determine whether genes associated with BCG in vitro and in vivo were enriched for A-to-I editing sites (Fig. 9E). When the sets of edited genes induced by BCG in vitro or proximal to a DMP in the MIS BAIR dataset were considered, both groups were enriched with editing signal in BCG-treated cells compared to Naïve-Mf, but only the in vivo DMP gene set was significantly more edited compared to the “background” set, which contains all edited transcripts (Fig. 9, E and F, and fig. S9D). The “induced by BCG in vitro” gene set was less edited than background under all conditions, but shows the largest boost in editing rate in Denmark-Mf and Bulgaria-Mf compared to Naïve-Mf, when controlling for average background gene editing across conditions (Fig. 9E). This indicates that BCG exposure leads to increased expression and subsequent A-to-I editing of target genes.

## DISCUSSION

TRIM is implicated in a range of inflammatory processes (*1*), with its involvement in the beneficial off-target effects of vaccines being of major clinical relevance (*19*). The beneficial off-target effects of the BCG vaccine persist for at least 1 year in infants (*17*) and are at least partly mediated through TRIM (*2*, *19*). The capacity of the BCG vaccine to induce TRIM has been shown in primary human adult monocytes in vitro (*27*) and in vivo (*19*) and involves functional reprogramming of bone marrow progenitors and monocytes in the circulation (*24*, *25*). Proof-of-principle clinical trials have shown that BCG-induced TRIM protects against viral infection in adults and the elderly (*19*, *21*), and current trials are testing BCG vaccination for protection of health care workers from COVID-19 (*22*, *39*).

Along with metabolic changes, epigenetic remodeling is a key process underlying the establishment of TRIM (*12*). Studies on various TRIM-inducing compounds have shown a role for histone modifications, nucleosome spacing, noncoding RNA, and DNA methylation in innate immune memory (*9*, *12*, *26*, *31*). Specific histone methyltransferases, such as G9a and Set7, have been shown to play major roles (*40*), while epigenetic inhibitors, such as the bromodomain inhibitor iBET, block both LPS-induced tolerance and β-glucan–induced TRIM (*26*). These chromatin remodeling events are mediated by TFs and, in turn, influence downstream gene expression (*41*).

In this study, we used an in vitro model of BCG-induced TRIM (*27*) to map the time-resolved epigenetic events that occur in human monocytes after direct exposure to BCG, and an in vivo model using the MIS BAIR clinical trial (*28*) to profile the long-term epigenetic alterations that remain more than 1 year after BCG vaccination of neonates. Monocytes directly exposed in vitro to BCG Denmark and BCG Bulgaria differentiate into trained macrophages with similar elevated cytokine production, gene expression, and epigenetic signatures (Fig. 1, B to E, and fig. S1), despite these vaccine strains potentially providing differing levels of protection against *M. tuberculosis* and nonspecific pathogens in vivo (*42*). This finding suggests that the primary driver of BCG-induced TRIM is IFNγ production and that SNPs and other genetic changes that have accumulated in these BCG vaccine strains do not prevent this activation. BCG exposure in vitro induced the expression of several hundred genes (Fig. 2B), some in a transient manner like LPS, marked by cytokine, chemokine, and TNF signaling genes, and a set of genes with a delayed response, marked by IRF motifs (Fig. 2D). This delayed up-regulated gene signature includes several human leukocyte antigen genes (table S1), including *HLA-DRA*, the expression of which has been linked to higher TNF production in macrophages (*43*).

An IFN-stimulated gene signature has previously been linked to BCG vaccination (*25*), and we identify previously unidentified BCG-induced genes such as *GBP1*, *GBP2*, and *GBP5* (guanylate-binding proteins; fig. S1), the latter of which is IFN-inducible and involved in inflammasome assembly and protection against viral infection (*44*, *45*). *GBP1* was also identified as more highly expressed in circulating monocytes from healthy donors following BCG vaccination, as well as in patients with bladder cancer following BCG instillation (*46*, *47*). IFNγ is specifically required for BCG-induced TRIM, while blocking IFNAR1 signaling with anifrolumab did not block training (Fig. 5). This is in contrast to the role for cell-intrinsic type I IFN (IFN-I) in β-glucan–induced TRIM, in which training of neutrophils was abolished in IFNAR1-deficient (*Ifnar1*^−/−^) mice (*48*). We note that the inhibition effect of anifrolumab in our model may not be complete, and therefore, a role for type I IFN signaling in BCG-induced TRIM cannot be ruled out.

Similar to gene expression changes, BCG-associated DNA methylation differences peaked at day 6 and were preceded by H3K27ac (Fig. 3), as previously described in monocytes (*26*) and dendritic cells (*32*). We then restimulated macrophages with LPS and observed enrichment of IFN, PRDM1, NFAT, and STAT motifs at genes that were trained for LPS response in BCG-Mf (Fig. 4). The IRF and PRDM1 signature was previously reported for genes trained in BMDMs derived from BCG-trained mice (*25*), while NFAT has been implicated in H3K4me3 marking of β-glucan–trained genes (*31*). Furthermore, the enrichment of viral response genes at trained genes (Fig. 4B) is in line with clinical data where BCG affords protection against viral infections (*21*).

BCG vaccination is associated with epigenetic reprogramming of circulating human monocytes in adults, with histone modification alterations shown at 1 month (*19*), and an open chromatin signature identified at 3 months following vaccination (*24*). This long-term TRIM-associated epigenetic signature in circulating cells can be traced to reprogramming of the hematopoietic progenitors in the bone marrow, leading to a myeloid bias (*24*, *25*). Here, we profiled DNA methylation, an environmentally sensitive covalent modification of DNA, 14 months after BCG vaccination in infants (Figs. 6 to 8). The BCG-vaccinated group can be separated from non–BCG-vaccinated controls based on their DNA methylation profile (Fig. 6B), indicating that the BCG-associated signature is maintained in monocyte progenitors, giving rise to epigenetically distinct monocytes. While several genes with an associated DMR in the BCG-vaccinated group showed higher expression in BCG-Mf in vitro (Fig. 8), this up-regulation was not accompanied by a methylation change in vitro. There was little overlap between in vivo and in vitro differential methylation, indicating that while the same pathways are altered by BCG in both settings, the chromatin context is different. Several large genomic regions (DMRs) (>2 kb in size and containing >10 DMPs) were identified in the BCG-vaccinated group, including at the promoter of *IFITM1* (fig. S8A), a gene involved in innate antiviral immunity (*49*). A recent single-cell transcriptome study identified *IFITM1* as differentially expressed in circulating monocytes 2 weeks and 3 months following BCG vaccination, as part of an IFNγ-mediated signaling signature (*50*). IFN response genes were enriched among those with a differentially methylated CpG site nearby (Fig. 6D), and the overall IFN signature is nicely illustrated by a DMR in the IFN-inducible promoter of the *ADAR1* gene (*51*, *52*). ADAR1 controls innate immune responses to RNA viruses through A-to-I editing of IFN-inducible RNA (*53*) and is implicated in a range of diseases including resistance to immune checkpoint blockade (*54*) and autoimmune disease (*55*). We show that A-to-I editing events are increased in BCG TRIM (Fig. 9), which is the first time this RNA modification has been linked to TRIM. However, we only show a correlation between elevated expression of the *ADAR1* gene and higher A-to-I rates in BCG-Mf and can therefore not rule out that other enzymes, such as ADAR2, may be involved in this process. The link between ADAR and inflammation in cancer and the role of BCG in treating bladder cancer may be interesting to explore in the future (*56*).

BCG remodels HSCs to promote myelopoiesis through IFNy (*25*), while *M. tuberculosis* reprograms HSCs via IFN-I to suppress myelopoiesis and impair TRIM to *M. tuberculosis* (*57*). Hence, the identification of DMRs at several IFN-responsive genes (Fig. 7A) supports these findings and suggests that the IFN-mediated HSC remodeling is carried in the chromatin for more than 1 year after vaccination and is therefore present in the circulating monocytes. Notably, the DMRs we identified at *ADAR1*, *IFITM1*, *MX2*, *IFI27*, and *IRF7* all show higher DNA methylation in the BCG-vaccinated group (Fig. 8A), but in the Kaufmann *et al.* (*25*) HSC data, the direction of expression in response to BCG varies from down-regulated to up-regulated (Fig. 8C). This highlights the need for further exploration, ideally in longitudinal samples from the same donor, to directly link HSC remodeling to long-term innate immune memory in peripheral circulating monocytes. Furthermore, we identify an IRF motif signature (IRF, PRDM1, and STAT1) in promoters of genes that are trained for LPS response by in vitro BCG exposure. This signature was also detected at genes that were trained for in vitro *M. tuberculosis* response in BMDMs exposed to BCG in vivo (*25*).

In conclusion, our data show that BCG-associated epigenetic remodeling in circulating infant monocytes is long-lived, lasting at least a year after neonatal vaccination. Neonates are the main population receiving the BCG vaccine, and therefore, this study is important because findings in adults do not always translate to children (*58*). The common IFN and viral response signature between in vitro BCG exposure and in vivo BCG vaccination, which are brought about by different mechanisms, direct exposure versus long-term programming of hematopoiesis, are in line with findings that BCG trained cells to respond well to viral infections (*19*, *21*).

## MATERIALS AND METHODS

### In vitro monocyte training protocol

Human primary monocytes were isolated from 50 ml of buffy coats from the Australian Red Cross Bloodline Service, Melbourne (ethics no. 18-10VIC-08). Peripheral blood mononuclear cells (PBMCs) were isolated using a Ficoll gradient (Merck, Sigma-Aldrich), followed by a hyper-osmotic Percoll separation and iso-osmotic Percoll separation. The TRIM protocol used has previously been published (*27*). Monocytes were differentiated into resting macrophages by in vitro culture in RPMI 1640 medium (Sigma-Aldrich) with 10% human serum (Sigma-Aldrich). The medium was supplemented with penicillin-streptomycin, 2 mM GlutaMAX, and 1 mM pyruvate (Gibco, Thermo Fisher Scientific). Monocytes were exposed to two different BCG vaccine strains: BCG Denmark (5 μg/ml; AJ Vaccines A/S, Copenhagen, Denmark) and BCG Bulgaria (5 μg/ml; BB-NCIPD Ltd., Sofia, Bulgaria). In addition, tolerance was induced using LPS (10 ng/ml; Sigma-Aldrich) and training using heat-inactivated *Candida albicans* [10^5^ colony-forming units (CFU)/ml] as a positive control. IFN signaling inhibition experiments were performed by culturing monocytes with BCG Denmark in the presence or absence of IL-18BP (10 μg/ml; Sigma-Aldrich) to block IFNγ or the IFNAR1 blocking antibody anifrolumab (10 μg/ml; ProteoGenix, France).

### MIS BAIR clinical trial participants

Participants in the in vivo study were a subset of 130 infants from the Melbourne Infant Study: BCG for the Prevention of Allergy and Infection (MIS BAIR) who were randomized to receive vaccination with BCG Denmark SSI 0.05 ml intradermally or no BCG vaccination within 10 days of birth. The MIS BAIR clinical trial has ethical approval from the Mercy Health Human Research Ethics Committee (HREC; no. R12-28) and Royal Children’s Hospital HREC (no. 33025). The inclusion and exclusion criteria for MIS BAIR have been previously described (*28*). MIS BAIR participants were invited to attend a 13-month study visit and provide a voluntary blood sample. All participants who provided a blood sample of sufficient volume for DNA methylation analysis were eligible for inclusion in this substudy. Exclusion criteria were acute illness in the preceding 48 hours, chronic illness, receipt of any vaccination in the preceding 7 days, and less than two doses of the routine primary scheduled vaccines.

### Isolation of circulating peripheral monocytes

PBMCs were isolated from whole blood collected from infants aged 12 to 24 months (median age, 14.5 months) in the MIS BAIR study and cryopreserved in liquid nitrogen. Cryo-preserved PBMCs were then thawed and prepared for sorting using the BD FACSAria Fusion. Monocytes were gated on CD14 and CD16 expression from the CD3^−^CD19^−^CD56^−^HLADR^+^ fraction. Classical (CD14^+^CD16^−^), intermediate (CD14^+^CD16^+^), and nonclassical (CD14^−^CD16^+^) monocytes were sorted and pooled. Monocytes were then lysed and cryo-preserved before DNA isolation.

### RNA sequencing

Stimulated monocytes were collected at various time points using TRIzol (Invitrogen, Thermo Fisher Scientific) and stored at −80°C until required. Total RNA was extracted from cells using the RNeasy Mini RNA Extraction Kit (Qiagen, The Netherlands), with additional on-column deoxyribonuclease I (Qiagen) treatment. RNA quality [RNA integrity number (RIN)] scores were determined using the RNA TapeStation System (Agilent). Libraries were prepared by the Victorian Clinical Genetic Services (VCGS) Sequencing Service (Melbourne, Australia) using the TruSeq Stranded mRNA Kit (Illumina). Libraries were sequenced paired-end on NovaSeq 6000 (Illumina) at ~20 million reads per sample, with 2 × 150 bp read lengths.

### ChIP sequencing

ChIP-seq was performed using the IHEC BLUEPRINT protocol, as published previously (*26*). Briefly, treated cells were fixed/crosslinked for 10 min with 1% formaldehyde (Sigma-Aldrich) and then resuspended in lysis buffer containing 1% SDS at a concentration of 25 × 10^6^ cells/ml. Chromatin shearing was performed using Covaris sonicator (S220) with the following settings: duty factor (DF), 5%; peak incident power (PIP), 140 W; cycles per burst (CB), 200 for 5 min. ChIP was performed using maximum 1 μg of chromatin with 1 μg of H3K27ac antibody (pAb-196-050t, Diagenode) and incubated overnight at 4°C with rotation. Protein A/G magnetic Dynabeads (Life Technologies) were added to the chromatin/antibody mix and rotated for 60 min at 4°C. The bead-antibody-protein-DNA complex was then extensively washed for total five times with low- and high-salt wash buffers at 4°C for 5 min each. After washing, chromatin was eluted off beads in elution buffer. Supernatant was collected, 8 μl of 5 M NaCl and 3 μl of proteinase K were added, and samples were de-crosslinked by incubating for 4 hours on a 65°C thermoshaker (Eppendorf). Last, samples were purified using the MinElute PCR Purification Kit (Qiagen). Detailed protocols can be found on the Blueprint website (https://www.blueprint-epigenome.eu/UserFiles/File/Protocols/Histone_ChIP_July2014.pdf). Library preparation was performed using the KAPA HyperPrep Kit (Roche) and sequenced paired-end on NovaSeq 6000 (Illumina) at ~30 million reads per sample, with 2 × 150 bp read length.

### DNA methylation EPIC array

Genomic DNA (500 ng) was isolated using a QIAamp DNA Micro kit (Qiagen), randomized on 96-well plates, and sent to the Erasmus MC Human Genotyping Facility (HuGe-F, The Netherlands) for sodium bisulfite treatment and genome-wide methylation analysis using Illumina Infinium MethylationEPIC BeadChips (“EPIC array”). The EPIC array measures DNA methylation level at more than 850,000 CpG sites (referred to as “EPIC probes”) and covers all gene promoters, gene bodies, and ENCODE-assigned distal regulatory elements. Raw IDAT files were used for downstream analysis.

### Cytokine production measurements

BCG-treated monocytes were cultured into macrophages in 96-well flat-bottom plates, and supernatants were collected at day 1 (24 hours of BCG exposure) and day 7 after overnight LPS restimulation and stored at −20°C. Supernatants from monocyte cultures were thawed and prepared for cytokine and chemokine quantification using the Bio-Plex Pro Human Cytokine 48-Plex Screening Panel (Bio-Rad) according to the manufacturer’s instructions. Data were acquired on the Bio-Plex 200 System. Of the 48 total cytokines, 44 were detected within the detectable range. Statistical analysis was performed using limma with a model containing the stimulation group and donor. TNF and IL-6 were also measured using an enzyme-linked immunosorbent assay (ELISA) kit according to the manufacturer’s protocol (R&D Systems). Supernatants were diluted 1:10 for IL-6 and 1:20 for TNF. For cytokine production assays, the differences between groups were analyzed using the Wilcoxon signed-rank test. The level of significance was defined as *P* < 0.05.

### Differential gene expression analysis

To infer gene expression levels, RNA-seq reads were aligned to hg19 human transcriptome using Bowtie (*59*). Quantification of gene expression was performed using MMSEQ (*60*). Statistical analysis was performed using DESeq2 (*61*), with pairwise comparisons performed across time (d0 v 4h v d1 v d6 v d6 + LPS) or between exposure (time-matched RPMI v BCG Denmark v BCG Bulgaria), with *P* < 0.05, FC > 2.5, Reads Per Kilobase of transcript, per Million mapped reads (RPKM) ≥ 5 used to determine a significant change in expression. Differential gene lists from all comparisons were then merged, and the combined list of differential genes was used for plotting.

### RNA editing quantification and filtering

RNA-seq reads were mapped to the human reference genome (GRCh37.75) using the STAR aligner (*62*) in two-pass mode. Mapped reads were deduplicated using PicardTools MarkDuplicates (broadinstitute.github.io/picard/), and candidate RNA editing sites were identified using JACUSA software v1.3.0 (*63*) in strand-specific mode, with D, S, and Y arguments used to filter out variants identified within 6 bp of the start or end of RNA reads, within 6 bp of an indel or splice site, or within 7 bp of homopolymeric read sequence, respectively. The JacusaHelper R package was used to import variant calls with test statistic values ≥ 1.56 and total read coverage ≥ 10. The minimal alternate (i.e., edited) allele depth was set to three. Common genomic SNPs were discarded, and only A>G variants detected in at least 3 of 19 samples were retained for further analysis. Sites were intersected with those catalogued in the REDIportal atlas (*64*), which incorporates the RADAR (*65*) and GTEx human tissue RNA editing databases (*66*). Sites with insufficient coverage were distinguished from transcribed reference alleles (i.e., unedited sites) using the samtools depth module (*67*). Only sites catalogued in the REDIportal atlas were considered in downstream analysis. A total of 4205 Alu sites were transcribed in all and edited in ≥3 samples, while 314 Alu sites were edited in all samples (fig. S9, B and C).

### RNA editing statistical testing

Using 314 sites that were edited in all conditions from all donors, the Alu editing index (AEI) for each sample was calculated as the sum of edited reads divided by the sum of transcribed reads. The change in AEI between each condition relative to RPMI-treated cells was estimated using the R lm() function, controlling for donor ID. To test differences in editing of gene sets between conditions, the total number of reference and alternative (edited) alleles was first summed for each gene in each sample. Genes were grouped into predefined sets (background, ex_vivo, in_vivo_dmp, and in_vivo_dmr signature genes), and differential editing was tested in each gene set using a generalized linear model with a binomial distribution in the R glm() function, controlling for donor ID. This has the advantage of incorporating the total reference and alternative allele counts, and thus the total read depth per site. Last, we used the same glm approach to test differential editing of individual genes in which editing sites were covered in at least six samples. Multiple testing was controlled using a false discovery rate of 0.01. Data were filtered, summarized, and plotted using the tidyverse suite of R packages.

### ChIP-seq data analysis

ChIP-sequencing reads were aligned to human genome assembly hg19 [National Center for Biotechnology Information (NCBI) version 37] using bwa (*68*). BAM files were first filtered to remove the reads with mapping quality less than 15 (*67*), followed by fragment size modeling using phantompeakqualtools (*69*). MACS2 was used to call peaks using the default (narrow) setting for H3K27ac and broad setting for H3K4me1 (*70*). Data (histone modification reads/peak) were normalized using the R package DESeq2 (*61*), and then pairwise comparisons were performed. Differential peaks were identified as *P* < 0.05, FC > 2, reads/peak > 50, using pairwise comparisons. Peaks were then merged into one file, which was used for plotting and *k*-means clustering to identify dynamics over time. The quality of the ChIP-seq data was visualized by making bigwig files using deepTools (*71*) in the UCSC Genome browser.

### DNA methylation analysis

Raw IDAT files were processed and analyzed using minfi packages for R (*72*). Samples were checked for quality, and those with a mean detection *P* value of >0.01 were removed. Data were normalized for both within and between array technical variation using SWAN (subset-quantile within array normalization) (*73*). Probes with poor average quality scores (detection *P* > 0.01), those associated with SNPs (minor allele frequency > 0%), and cross-reactive probes were removed from further analysis. This left a total of 771,000 probes for downstream analysis. Differential methylation analysis by linear regression modeling was performed using limma (*74*). Technical confounders were identified using principal components analysis and were incorporated in the analysis models as required. DMPs were those that showed an adjusted *P* value of <0.05 (Benjamini-Hochberg) and a change in methylation (delta beta or Δβ) of 0.05 (>5%). DMRs were identified using the DMRcate tool (*75*). DMPs were assigned to the nearest gene within 1 Mb using the GREAT tool (*76*); however, for gene ontology analysis, we restricted gene lists to those with a DMP within 5 kb of their transcription start site.

### Gene ontology and motif enrichment analysis

Gene ontology analysis on differentially expressed genes was performed using HOMER (*77*). Gene ontology on genes with DMRs in their promoters was performed using HOMER and GREAT (*76*) and, in the case of DMRs, confirmed using GOmeth (*78*). KEGG (Kyoto Encyclopedia of Genes and Genomes) pathways and Biological Processes were ranked by *P* value, and the top unique terms were plotted. Motif enrichment was performed using HOMER (*77*). For differentially expressed gene lists, we measured promoter motif abundance using the “findMotifs” tool, while for DMRs and dynamic enhancers, we used the “findMotifsGenome” tool. For each motif scan, we used the full list of differentially expressed genes, except for the LPS restimulation where we used a sliding window approach. Genes were ranked by their response to LPS in BCG-trained macrophages, and then windows of 60 genes were scanned for motif enrichment.

### Integration of publicly available TRIM data

RNA-seq data from human in vitro trained immunity models were downloaded from the following NCBI Gene Expression Omnibus accession numbers: GSE166238 for oxLDL-trained macrophages, GSE110947 for heme-trained macrophages, and GSE85243 for β-glucan–trained macrophages. Data from mouse HSCs exposed to BCG were downloaded from GSE98600. The expression values for gene sets of interest, for example, those associated with monocyte-to-macrophage differentiation, or specific genes, for example, those near a BCG-associated DMP, were extracted from these public datasets.

### Training QTL mapping in 300BCG

To investigate whether genetic variation at genes near DMPs influences BCG-induced TRIM, QTL mapping was studied in a cohort of healthy volunteers vaccinated with BCG [300BCG cohort, part of Human Functional Genomics Project (HFGP)] (*38*). The cohort consists of 321 healthy Dutch individuals of western European descent (139 men and 182 women; age range, 18 to 71 years) vaccinated with BCG (InterVax). The 300BCG study was approved by the Arnhem-Nijmegen Medical Ethical Committee, NL58553.091.16. Genetic data from the HFGP are subjected to a data access agreement, which is available at www.humanfunctionalgenomics.org. Inclusion of volunteers and experiments was conducted according to the principles expressed in the Declaration of Helsinki. All volunteers gave written informed consent before any material was taken. Before vaccination, and 2 weeks and 3 months after vaccination, blood was drawn and PBMCs were isolated. PBMCs from 287 individuals were stimulated with heat-killed *S. aureus* (5 × 10^6^ CFU/ml), as previously described (*38*). DNA samples of individuals were genotyped using the commercially available SNP chip, Infinium Global Screening Array MD v1.0 from Illumina. Cis cQTLs that influence cytokine release in response to *S. aureus* in BCG-vaccinated individuals were identified (table S7). The analysis was performed genome-wide using a linear model with age and sex as covariates (*38*). The resulting cQTLs were overlapped with genes of interest, DMPs, and DMRs using bedtools (*79*).

## Notes

View/request a protocol for this paper from *Bio-protocol*.
