# Supplementary material for: Neonatal BCG vaccination is associated with a long-term DNA methylation signature in circulating monocytes
Source: Sci Adv. 2022 Aug 5;8(31):eabn4002. doi: 10.1126/sciadv.abn4002 (PMC9355358; doi:10.1126/sciadv.abn4002)
Supplement: Supplementary file 1 — Figs. S1 to S9 [file sciadv.abn4002_sm.pdf]

Science Advances
AAAS

Supplementary Materials for

Neonatal BCG vaccination is associated with a long-term DNA methylation signature in circulating monocytes

Samantha Bannister *et al.*

Corresponding author: Boris Novakovic, boris.novakovic@mcri.edu.au; Nigel Curtis, nigel.curtis@mcri.edu.au

Sci. Adv. **8**, eabn4002 (2022)
DOI: 10.1126/sciadv.abn4002

The PDF file includes:

Figs. S1 to S9
Legends for tables S1 to S8

Other Supplementary Material for this manuscript includes the following:

Tables S1 to S8

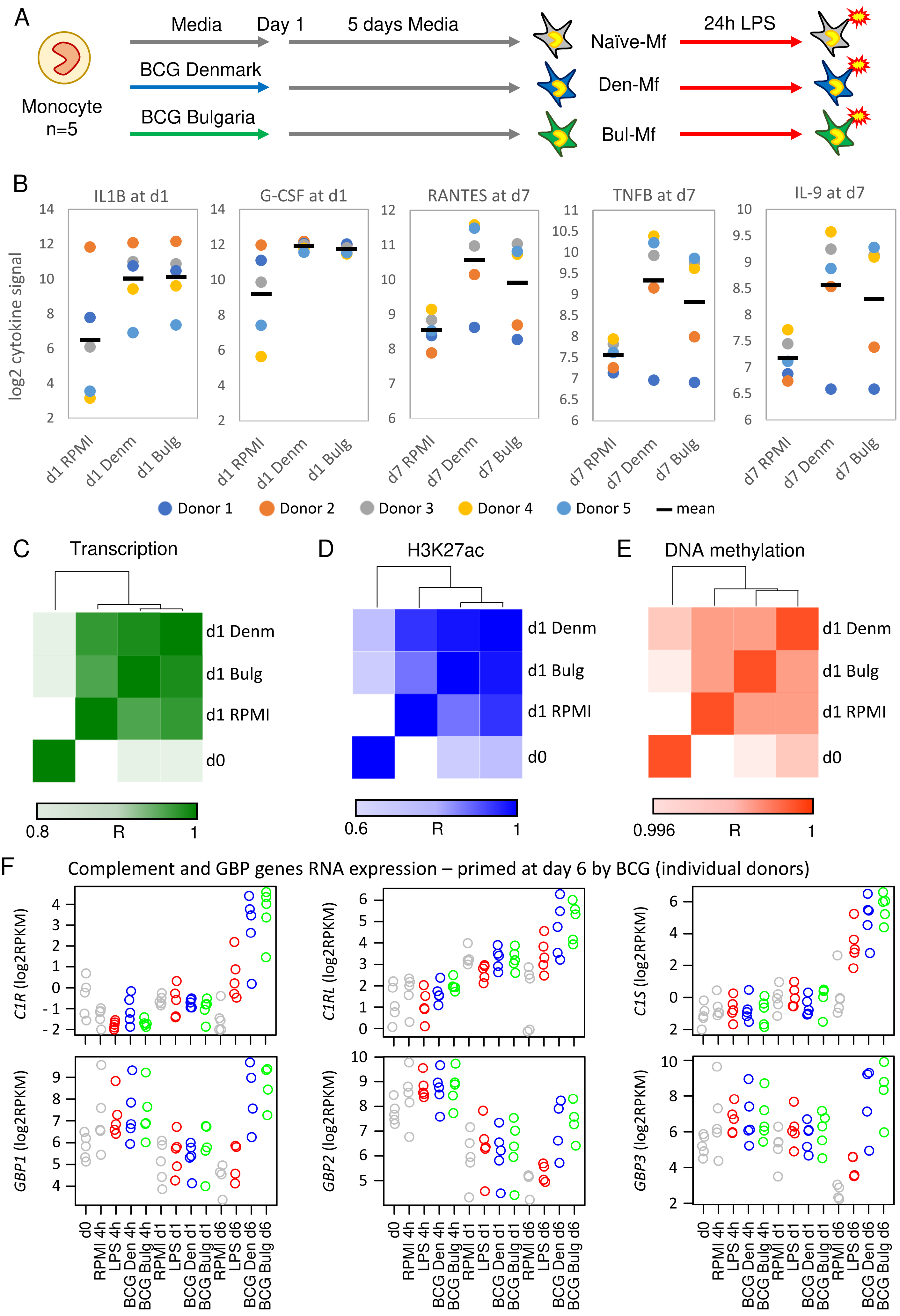
A
Monocyte
n=5
Media
Day 1
5 days Media
BCG Denmark
BCG Bulgaria
Naïve-Mf
Den-Mf
Bul-Mf
24h LPS
B
log2 cytokine signal
IL1B at d1
G-CSF at d1
RANTES at d7
TNFB at d7
IL-9 at d7
d1 RPMI
d1 Denm
d1 Bulg
d7 RPMI
d7 Denm
d7 Bulg
Donor 1
Donor 2
Donor 3
Donor 4
Donor 5
mean
C
Transcription
D
H3K27ac
E
DNA methylation
d1 Denm
d1 Bulg
d1 RPMI
d0
0.8
R
1
0.6
0.996
F
Complement and GBP genes RNA expression – primed at day 6 by BCG (individual donors)
C1R (log2RPKM)
C1RL (log2RPKM)
C1S (log2RPKM)
GBP1 (log2RPKM)
GBP2 (log2RPKM)
GBP3 (log2RPKM)
d0
RPMI 4h
LPS 4h
BCG Den 4h
BCG Bulg 4h
RPMI d1
LPS d1
BCG Den d1
BCG Bulg d1
RPMI d6
LPS d6
BCG Den d6
BCG Bulg d6

Figure S1. <u>*Related to Figure 1.*</u> ***Two different strains of BCG vaccine induce similar transcriptional, epigenetic and cytokine signatures.*** **A.** Experimental set-up for the *in vitro* TRIM protocol, including HKCA as a positive TRIM control, LPS as a tolerance control. **B.** Bar plot showing TNF and IL-6 release at day 6 for each donor compared to naïve-Mf control. LPS-Mf show a tolerised response relative to Naïve-Mf, while HKCA-Mf and BCG-Mf show a trained response. Correlation matrix heatmap of day 0 and day 1 samples based on BCG associated **C.** gene expression, **D.** H3K27ac and **E.** DNA methylation change. H3K27ac showed the largest variation (R 0.6 – 1), while DNA methylation showed the smallest variation (R 0.996 – 1) within the first 24 hours. **F.** Dot plots showing RNA expression for specific genes of interest in individual donors as log2RPKM values. The complement genes *C1R, C1RL, C1S*, and Guanylate-Binding Proteins, *GBP1, -2* and *-3* all show higher expression in BCG day 6 samples.

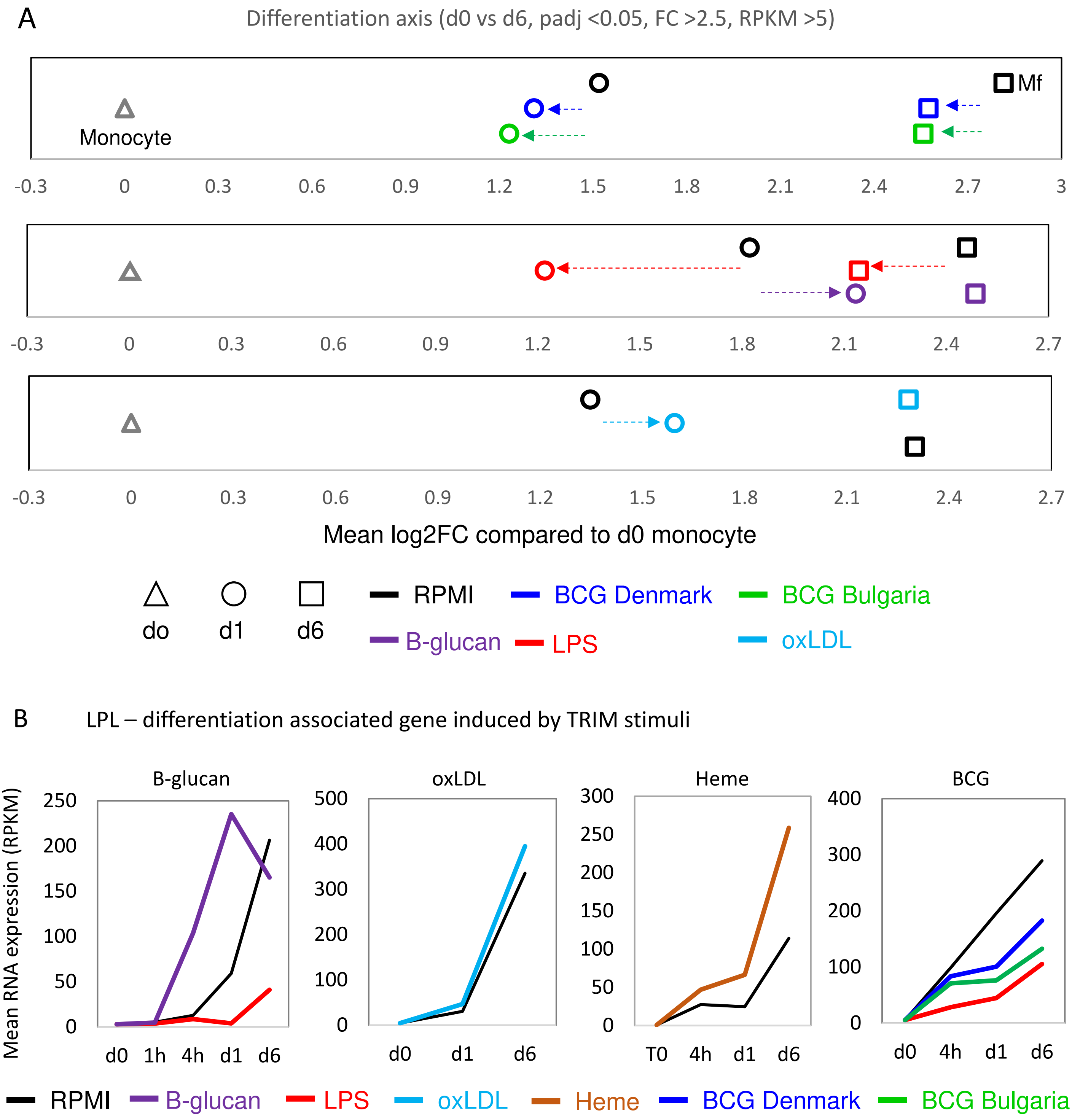

A
Differentiation axis (d0 vs d6, padj <0.05, FC >2.5, RPKM >5)
Mf
Monocyte
-0.3
0
0.3
0.6
0.9
1.2
1.5
1.8
2.1
2.4
2.7
3
Mean log2FC compared to d0 monocyte
do
d1
d6
RPMI
BCG Denmark
BCG Bulgaria
B-glucan
LPS
oxLDL
B
LPL – differentiation associated gene induced by TRIM stimuli
B-glucan
oxLDL
Heme
BCG
Mean RNA expression (RPKM)
d0
1h
4h
d1
d6
T0
RPMI
B-glucan
LPS
oxLDL
Heme
BCG Denmark
BCG Bulgaria

Figure S2. *Related to Figure 2*. ***BCG induced Trained immunity involves a unique transcriptomic profile, unrelated to differentiation.*** **A**. Dot plot of mean log2 fold change relative to day 0 monocytes, based on monocyte-to-macrophage differentiation associated gene expression in each dataset (p.adj <0.05, FC >2.5, RPKM >5). (**i**) The first panel shows the current BCG experiment, based on 1,734 differentiation-associated Genes, with monocytes (triangle), day 1 RPMI (black circle) and day 6 RPMI macrophage (black square) distributed from left to right. BCG exposure shifts day 1 (blue and green circles) and day 6 (blue and green squares) expression profile towards the left, attenuating differentiation. (**ii**) B-glucan accelerates differentiation-associated gene expression, while LPS attenuates the process. Plot based on 839 genes. (**iii**) oxLDL accelerates the differentiation-associated gene expression at day 1 (light blue circle). Plot based on 1,428 genes. **B**. Expression of lipoprotein lipase (*LPL*) in monocytes over time exposed to B-glucan, LPS, Heme, oxLDL and BCG Denmark and BCG Bulgaria. *LPL* expression increases with differentiation, with TRIM stimuli accelerating the upregulation, except for BCG, which attenuates *LPL*.

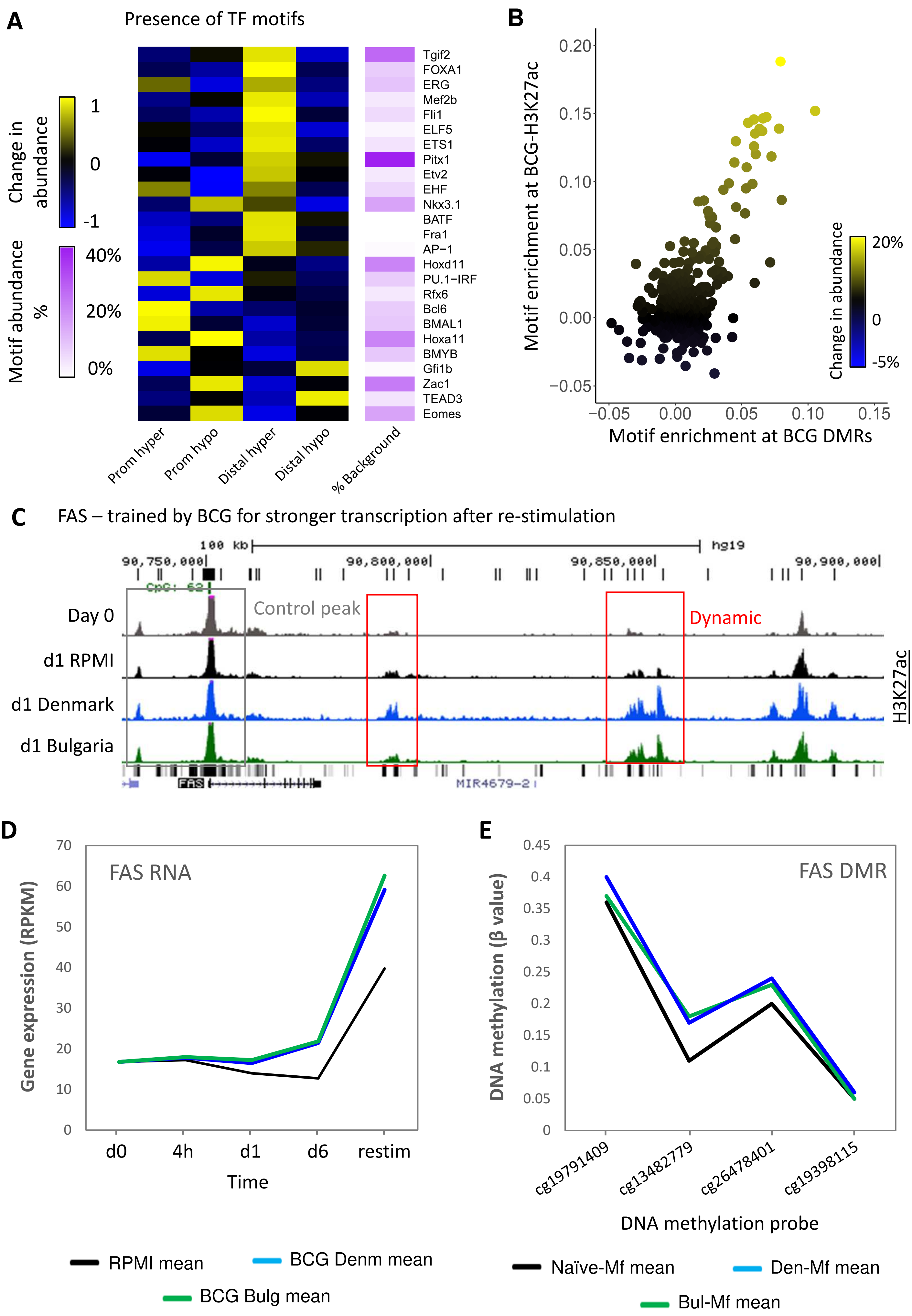

A
Presence of TF motifs
Change in abundance
1
0
-1
Motif abundance %
40%
20%
0%
Tgif2
FOXA1
ERG
Mef2b
Fli1
ELF5
ETS1
Pitx1
Etv2
EHF
Nkx3.1
BATF
Fra1
AP−1
Hoxd11
PU.1−IRF
Rfx6
Bcl6
BMAL1
Hoxa11
BMYB
Gfi1b
Zac1
TEAD3
Eomes
Prom hyper
Prom hypo
Distal hyper
Distal hypo
% Background
B
Motif enrichment at BCG-H3K27ac
Motif enrichment at BCG DMRs
Change in abundance
20%
0
-5%
C
FAS – trained by BCG for stronger transcription after re-stimulation
100 kb
hg19
90,750,000
90,800,000
90,850,000
90,900,000
CpG: 62
Day 0
d1 RPMI
d1 Denmark
d1 Bulgaria
Control peak
Dynamic
H3K27ac
FAS
MIR4679-2
D
FAS RNA
Gene expression (RPKM)
d0
4h
d1
d6
restim
Time
RPMI mean
BCG Denm mean
BCG Bulg mean
E
FAS DMR
DNA methylation (β value)
cg19791409
cg13482779
cg26478401
cg19398115
DNA methylation probe
Naïve-Mf mean
Den-Mf mean
Bul-Mf mean

Figure S3. *Related to Figure 3*. ***Coordinated remodelling of H3K27ac, DNA methylation and gene expression.*** **A.** Motif scanning identified distinct differences between promoter and distal DMRs and those that show increased (hyper) methylation in response to BCG. **B.** Scatter plot showing high correlation between motif enrichment at BCG-Induced H3K27ac regions and BCG-associated DMRs. **C.** Map of *FAS* gene, which shows a trained LPS response in BCG-Mf, with H3K27ac signal tracks. Downstream distal elements show increase in H3K27ac signal 24 hours after BCG Denmark and BCG Bulgaria exposure. D. Differentially methylated region (DMR) near the FAS gene and **E**. Expression of *FAS* over time in RPMI or BCG exposed monocytes, showing a trained profile in BCG-Mf.

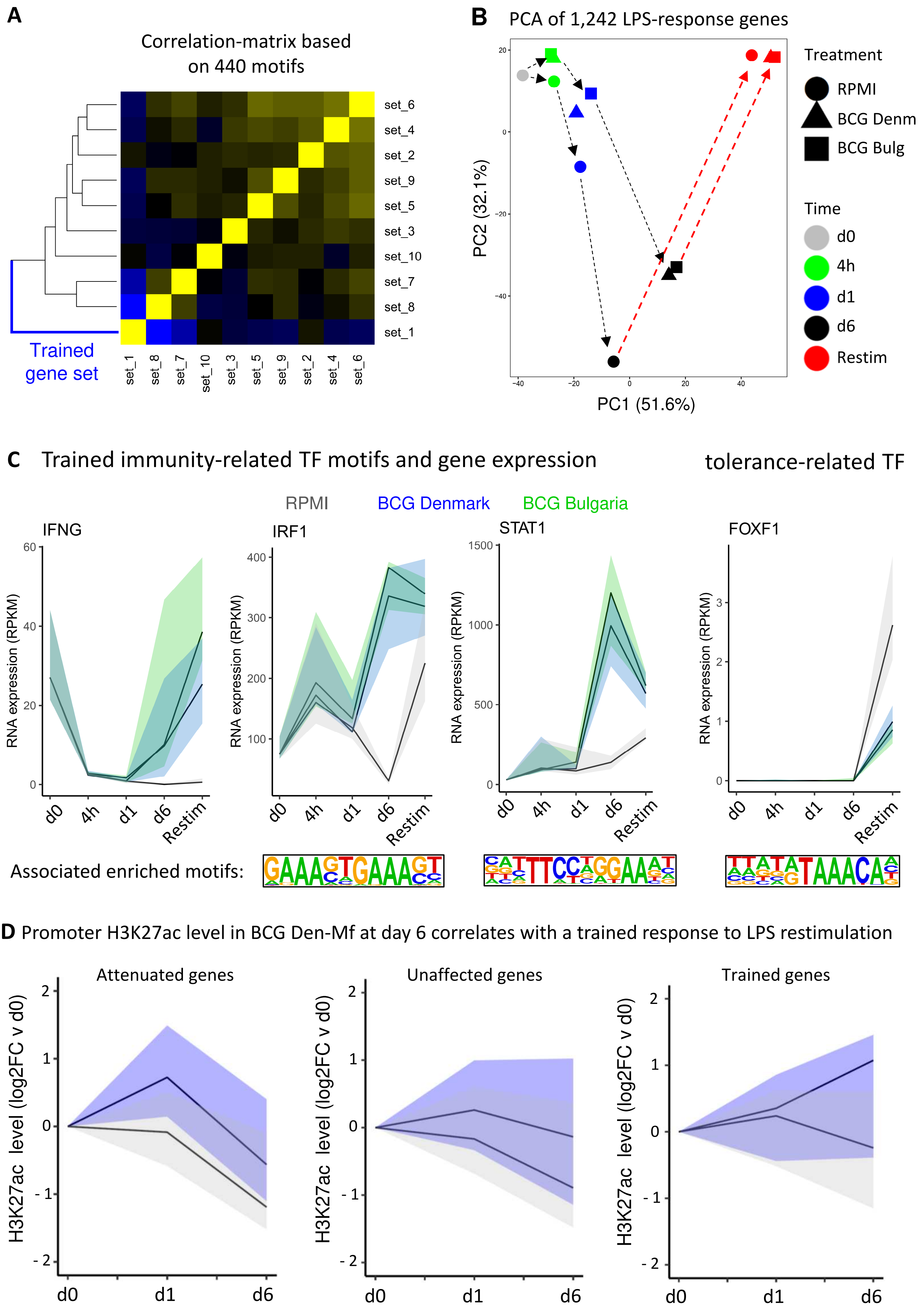
A
Correlation-matrix based on 440 motifs
set_6
set_4
set_2
set_9
set_5
set_3
set_10
set_7
set_8
set_1
Trained gene set
B
PCA of 1,242 LPS-response genes
PC1 (51.6%)
PC2 (32.1%)
Treatment
RPMI
BCG Denm
BCG Bulg
Time
d0
4h
d1
d6
Restim
C
Trained immunity-related TF motifs and gene expression
tolerance-related TF
RPMI
BCG Denmark
BCG Bulgaria
IFNG
IRF1
STAT1
FOXF1
RNA expression (RPKM)
d0
4h
d1
d6
Restim
Associated enriched motifs:
D Promoter H3K27ac level in BCG Den-Mf at day 6 correlates with a trained response to LPS restimulation
Attenuated genes
Unaffected genes
Trained genes
H3K27ac level (log2FC v d0)
d0
d1
d6

Figure S4. *Related to Figure 4*. ***Transcription Factor motif signature associated with LPS restimulation in BCG-Mf.*** **A.** Correlation matrix based on abundance of 440 TF motifs across gene sets induced by LPS at day 6. Genes were separated into 10 sets of 120 (out of total 1,242 genes in **Figure 4B**), with set_1 showing a trained LPS response in BCG-Mf and set_10 showing an attenuated LPS response in BCG-Mf. The trained gene set_1 clustered away from others, indicating that this set of genes have a unique motif signature. **B**. PCA plot of RPMI and BCG exposed cells over time, based on 1,242 LPS responsive genes at day 6. BCG-Mf show a slightly inflamed expression profile. **C**. Expression of genes encoding TFs associated with trained gene promoter motifs (*IRF1* and *STAT1*), and *FOXF1*, which has an enriched motif at attenuated genes. In addition to upregulated *IRF1* in BCG-Mf compared to RPMI-Mf, these cells also show higher expression of IFNγ. **D**. The 1,242 LPS responsive genes were separated into attenuated (100 genes), unaffected (946 genes) and trained (196 genes). Dynamic H3K27ac (10,329 peaks) within 5kb of the promoter region was plotted at these genes. At attenuated and unaffected gene promoters H3K27ac is elevated in BCG exposed monocytes at 24 hours. At trained genes, H3K27ac accumulates at day 6 in BCG-Mf. This indicates that the later H3K27ac accumulation (**Figure 3E**) is priming the trained transcriptional response to LPS restimulation in BCG-Mf.

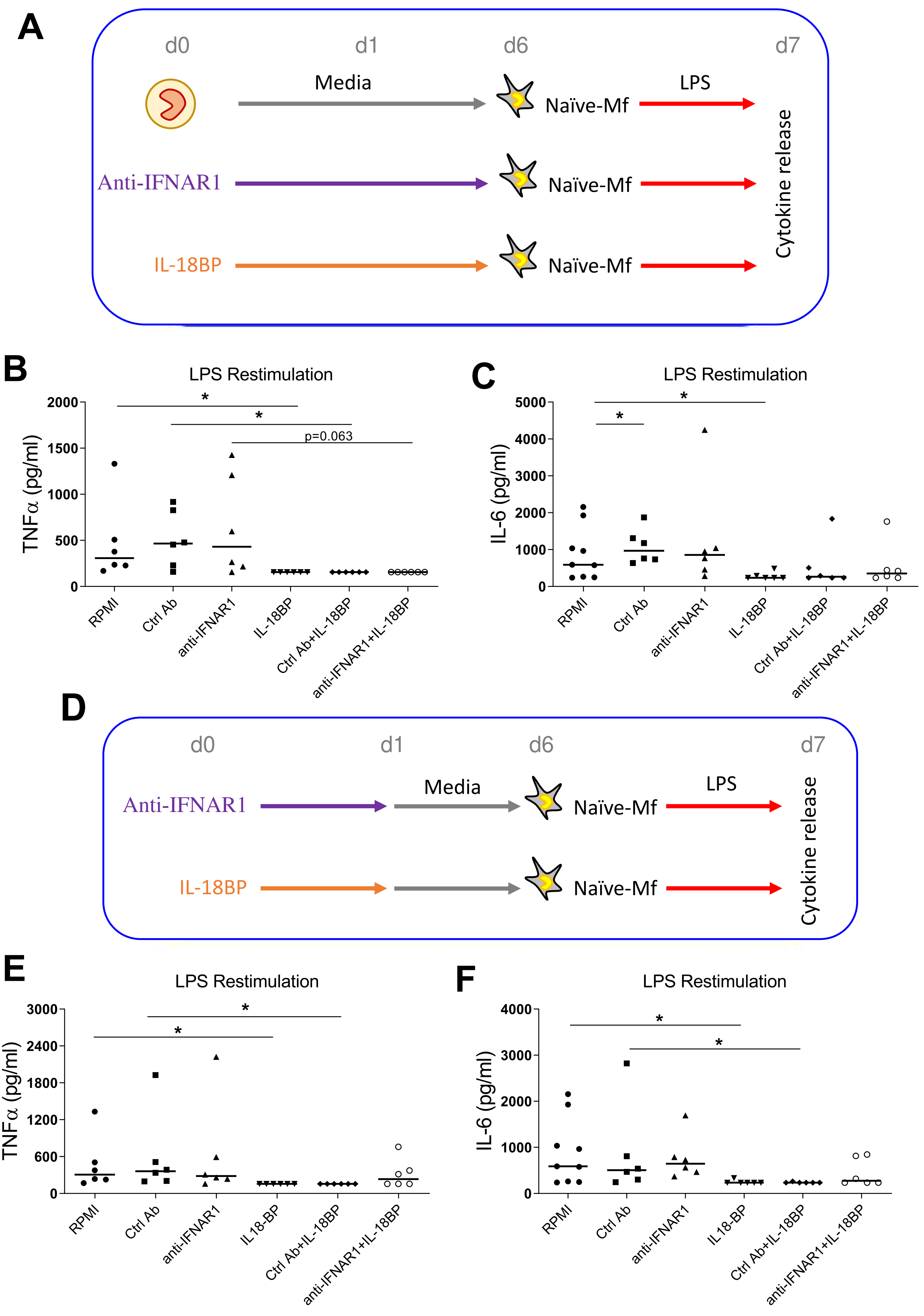
A
d0
d1
d6
d7
Media
Naïve-Mf
LPS
Anti-IFNAR1
Naïve-Mf
IL-18BP
Naïve-Mf
Cytokine release
B
LPS Restimulation
TNFα (pg/ml)
p=0.063
RPMI
Ctrl Ab
anti-IFNAR1
IL-18BP
Ctrl Ab+IL-18BP
anti-IFNAR1+IL-18BP
C
LPS Restimulation
IL-6 (pg/ml)
RPMI
Ctrl Ab
anti-IFNAR1
IL-18BP
Ctrl Ab+IL-18BP
anti-IFNAR1+IL-18BP
D
d0
d1
d6
d7
Anti-IFNAR1
Media
Naïve-Mf
LPS
IL-18BP
Naïve-Mf
Cytokine release
E
LPS Restimulation
TNFα (pg/ml)
RPMI
Ctrl Ab
anti-IFNAR1
IL18-BP
Ctrl Ab+IL-18BP
anti-IFNAR1+IL-18BP
F
LPS Restimulation
IL-6 (pg/ml)
RPMI
Ctrl Ab
anti-IFNAR1
IL18-BP
Ctrl Ab+IL-18BP
anti-IFNAR1+IL-18BP

Figure S5. (Related to Figure 5) **A.** *In vitro* model to test the effect of IL-18BP and anti-IFNAR1 Ab on trained immunity. Monocytes exposed to anti-IFNAR1 Ab (purple line) and IL-18BP (orange line) were in the culture medium for the entire 6 days. At day 6, macrophages were exposed to LPS and cytokine release was measured 24 hours later (day 7). Dotplots showing cytokine release in pg/mL for **B.** TNF and **C.** IL6 at day 7 **D.** *In vitro* model of inhibitor co-stimulation for 24 hours only, followed by media for 5 days, before LPS restimulation. Dotplots showing cytokine release in pg/mL for **E.** TNF and **F.** IL6 production at day 7, 24 hours after LPS restimulation. n=6-9. *$p<0.05$.

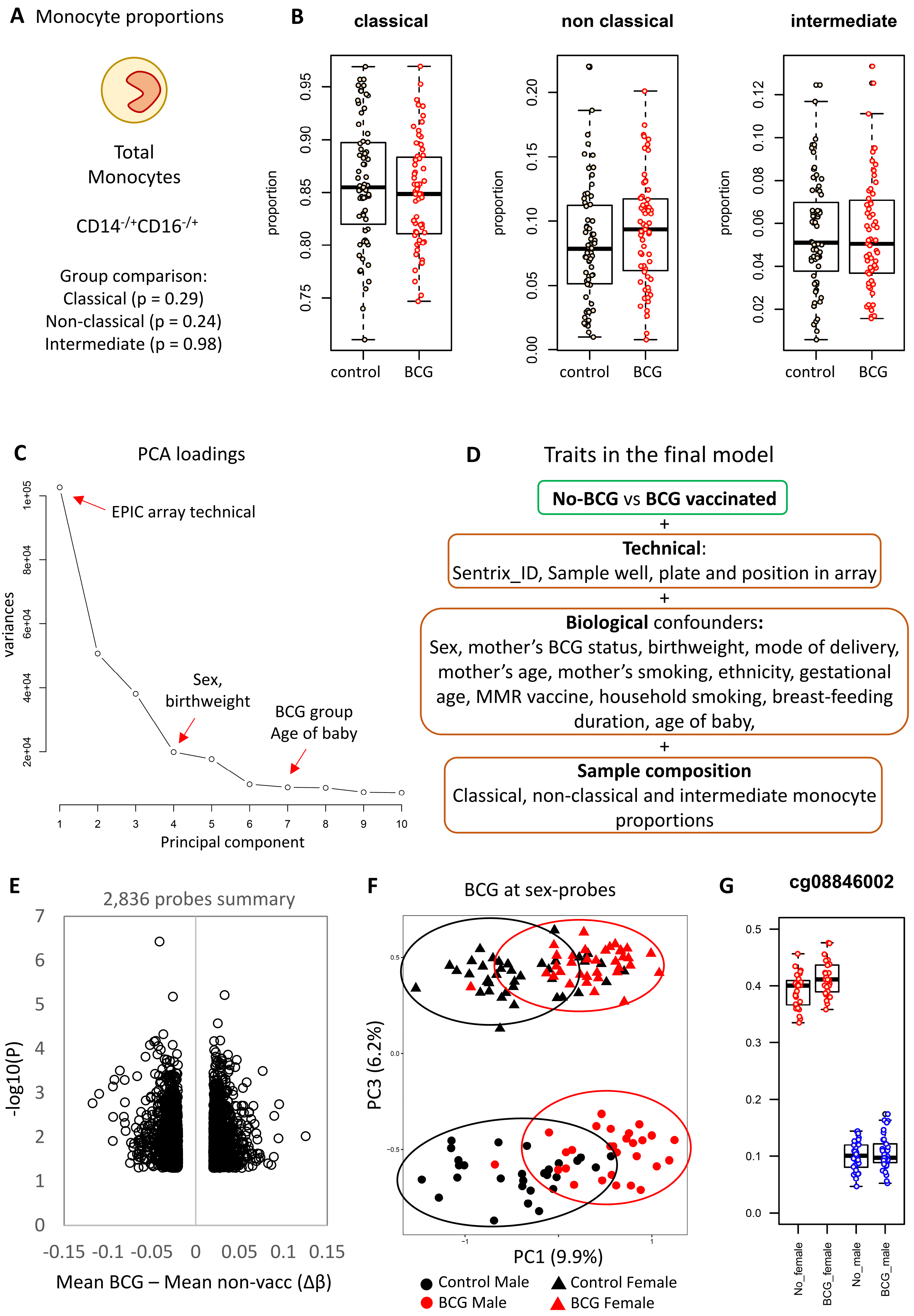
A Monocyte proportions
Total Monocytes
CD14-/+CD16-/+
Group comparison:
Classical (p = 0.29)
Non-classical (p = 0.24)
Intermediate (p = 0.98)
B
classical
non classical
intermediate
proportion
control
BCG
C PCA loadings
EPIC array technical
Sex, birthweight
BCG group Age of baby
variances
Principal component
D Traits in the final model
No-BCG vs BCG vaccinated
+
Technical:
Sentrix_ID, Sample well, plate and position in array
+
Biological confounders:
Sex, mother's BCG status, birthweight, mode of delivery, mother's age, mother's smoking, ethnicity, gestational age, MMR vaccine, household smoking, breast-feeding duration, age of baby,
+
Sample composition
Classical, non-classical and intermediate monocyte proportions
E
2,836 probes summary
-log10(P)
Mean BCG – Mean non-vacc (Δβ)
F
BCG at sex-probes
PC3 (6.2%)
PC1 (9.9%)
Control Male
Control Female
BCG Male
BCG Female
G
cg08846002
No_female
BCG_female
No_male
BCG_male

Figure S6. *Related to Figure 6*. ***Summary of the statistical model used to identify BCG-associated DNA methylation signature.*** **A.** P values for monocyte subtype proportions between the non-BCG-vaccinated and BCG-vaccinated groups, showing no difference. **B**. Boxplot of classical, intermediate, and non-classical monocyte proportions for individuals in non-BCG-vaccinated (black) and BCG-vaccinated (red) groups. **C**. PCA loadings, with primary contributors to variation for each principal component. **D**. Technical and biological co-variants included in the final statistical model. **E**. Volcano plot of 2,836 DMPs that were significant between non-BCG-vaccinated and BCG vaccinated groups ($p < 0.05$, dB >0.02). **F**. BCG effect is seen on the X chromosome, with clear separation of BCG and non-BCG-vaccinated groups on PC1, and separation by sex on PC3. This plot was generated using the same probes as Figure 6B, which shows PC1 v PC2. **G**. Boxplot of a BCG-DMP on the X chromosome.

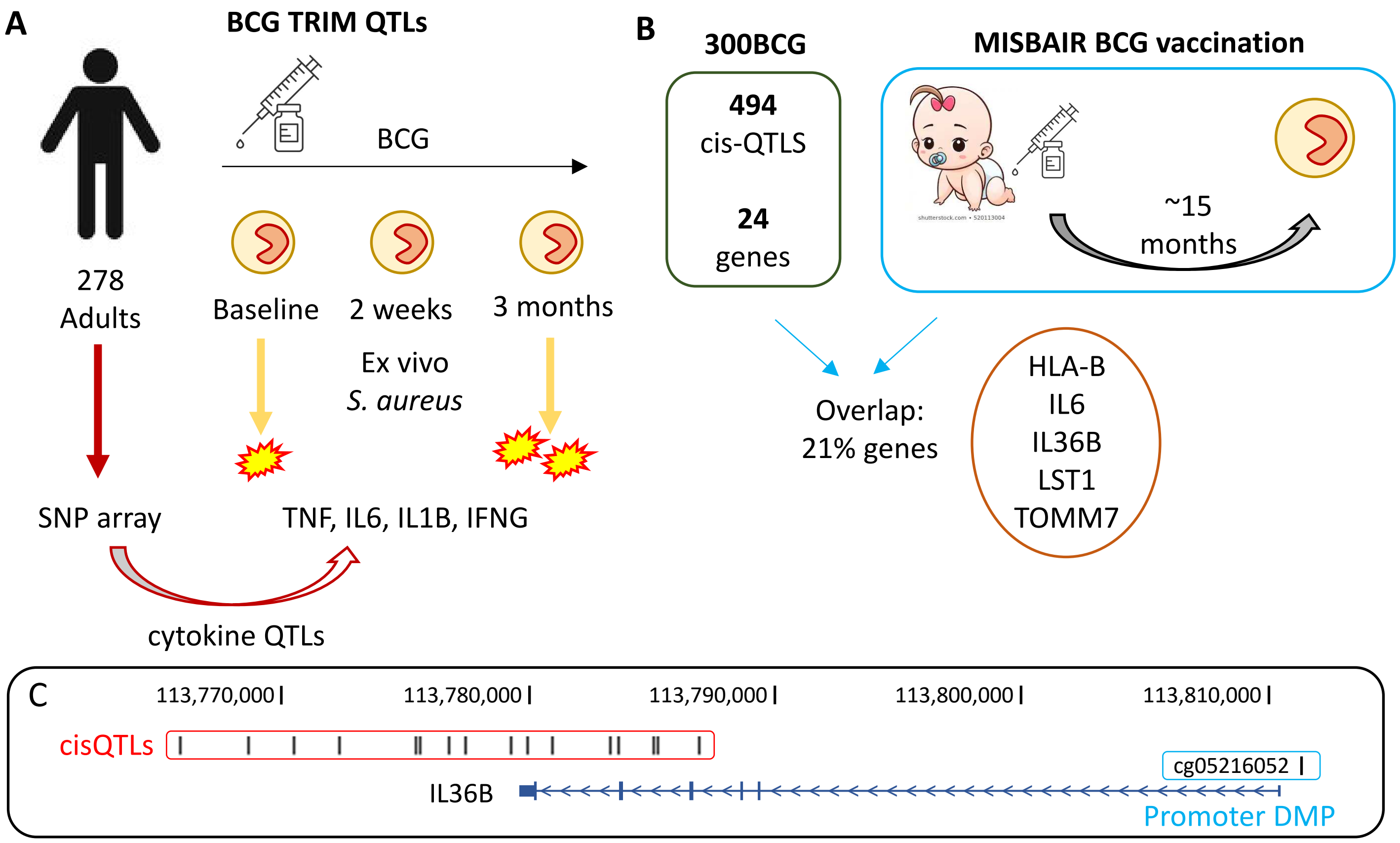
A
BCG TRIM QTLs
BCG
278
Adults
Baseline
2 weeks
3 months
Ex vivo
S. aureus
SNP array
TNF, IL6, IL1B, IFNG
cytokine QTLs
B
300BCG
494
cis-QTLS
24
genes
MISBAIR BCG vaccination
~15
months
Overlap:
21% genes
HLA-B
IL6
IL36B
LST1
TOMM7
C
113,770,000
113,780,000
113,790,000
113,800,000
113,810,000
cisQTLs
cg05216052
IL36B
Promoter DMP

Figure S7. Related to Figure 6. ***Overlap between cytokine QTLs upon BCG-induced TRIM and differential DNA methylation associated with BCG vaccination.*** **A**. Model for identification of cytokine-release associated quantitative trait loci (cytokine-QTLs) in the 300BCG cohort. **B.** In total, 24 genes were associated with a cytokine-QTL in trans, of which 5 were also associated with a DMP in the MIS-BAIR dataset. **C**. Map of the *IL36B* gene, which has a BCG-associated DMP in the promoter region and several cis cytokine-QTLs at the 3' end.

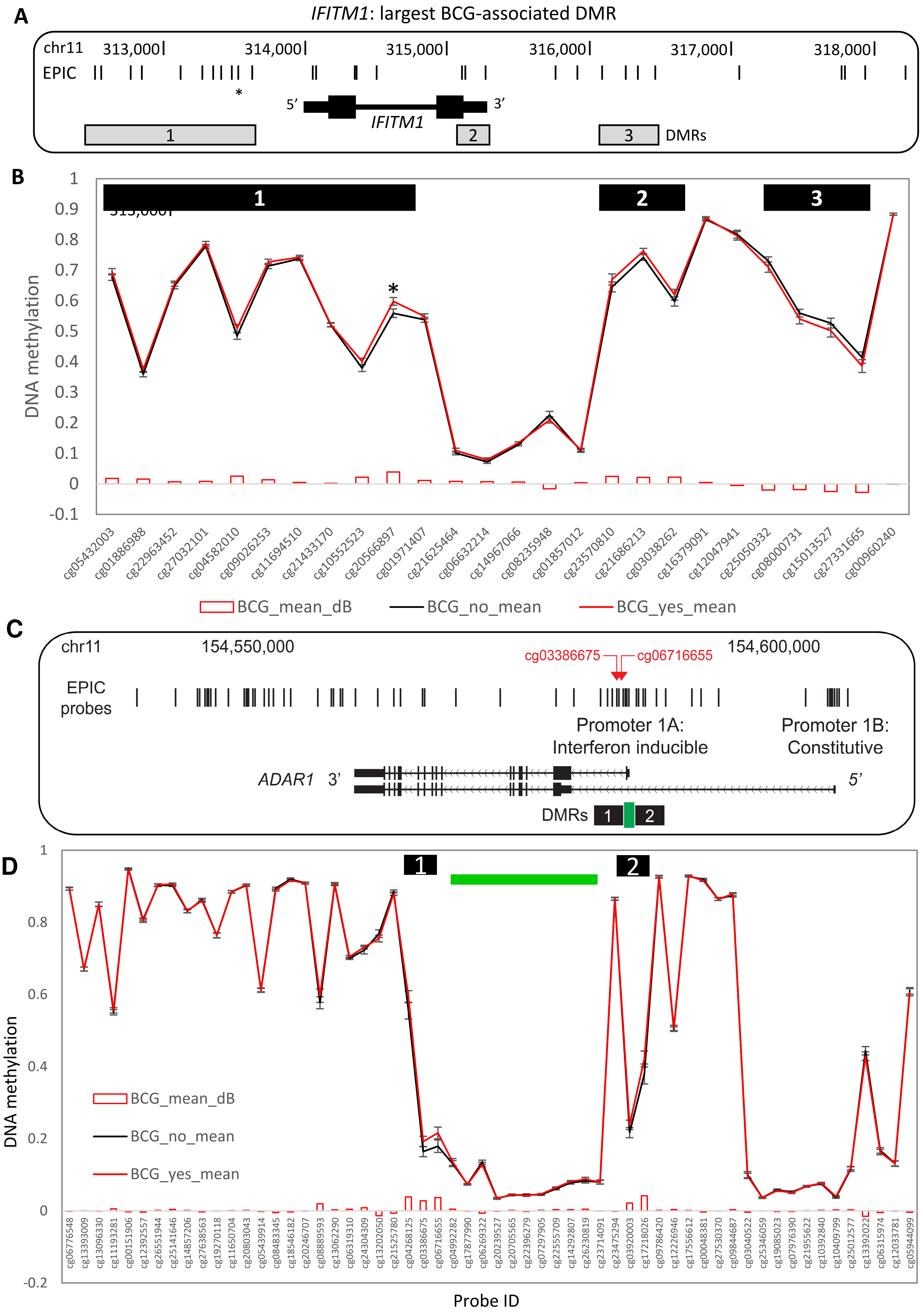

A
IFITM1: largest BCG-associated DMR
chr11
313,000
314,000
315,000
316,000
317,000
318,000
EPIC
5′
3′
IFITM1
DMRs
1
2
3
B
DNA methylation
1
0.9
0.8
0.7
0.6
0.5
0.4
0.3
0.2
0.1
0
-0.1
cg05432003
cg01886988
cg22963452
cg27032101
cg04582010
cg09026253
cg11694510
cg21433170
cg10552523
cg20566897
cg01971407
cg21625464
cg06632214
cg14967066
cg08235948
cg01857012
cg23570810
cg21686213
cg03038262
cg16379091
cg12047941
cg25050332
cg08000731
cg15013527
cg27331665
cg00960240
BCG_mean_dB
BCG_no_mean
BCG_yes_mean
C
chr11
154,550,000
154,600,000
cg03386675
cg06716655
EPIC probes
Promoter 1A: Interferon inducible
Promoter 1B: Constitutive
ADAR1
3′
5′
DMRs
1
2
D
DNA methylation
1
0.8
0.6
0.4
0.2
0
-0.2
BCG_mean_dB
BCG_no_mean
BCG_yes_mean
cg06776548
cg13393009
cg13096330
cg11193281
cg00151906
cg12392557
cg26551944
cg25141646
cg14857206
cg27638563
cg19270118
cg11650704
cg20803043
cg05439914
cg08483345
cg18546182
cg20246707
cg08889593
cg13062290
cg06319310
cg24304309
cg13202050
cg21525780
cg04268125
cg03386675
cg06716655
cg04992282
cg17877990
cg06269322
cg20239527
cg20705565
cg22396279
cg07297905
cg22555709
cg14292807
cg26230819
cg23714091
cg23475294
cg03920003
cg17218026
cg09786420
cg12226946
cg17556612
cg00048381
cg27530370
cg09844687
cg03040522
cg25346059
cg19085023
cg07976390
cg21955622
cg10392840
cg10409799
cg25012577
cg13392022
cg06315974
cg12033781
cg05944099
Probe ID

Figure S8. Related to Figure 7. **A.** Map of the *IFITM1* gene locus, showing three DMRs located upstream and downstream of the gene. **B.** DNA methylation is higher in the BCG group within DMR1 and lower in DMR2 and DMR3. **C.** Extended map of the *ADAR1* gene locus showing **D.** consistent DNA methylation across the ADAR1 gene, except at two DMRs that occur at either side of a CpG Island, with higher methylation in the BCG-vaccinated group.

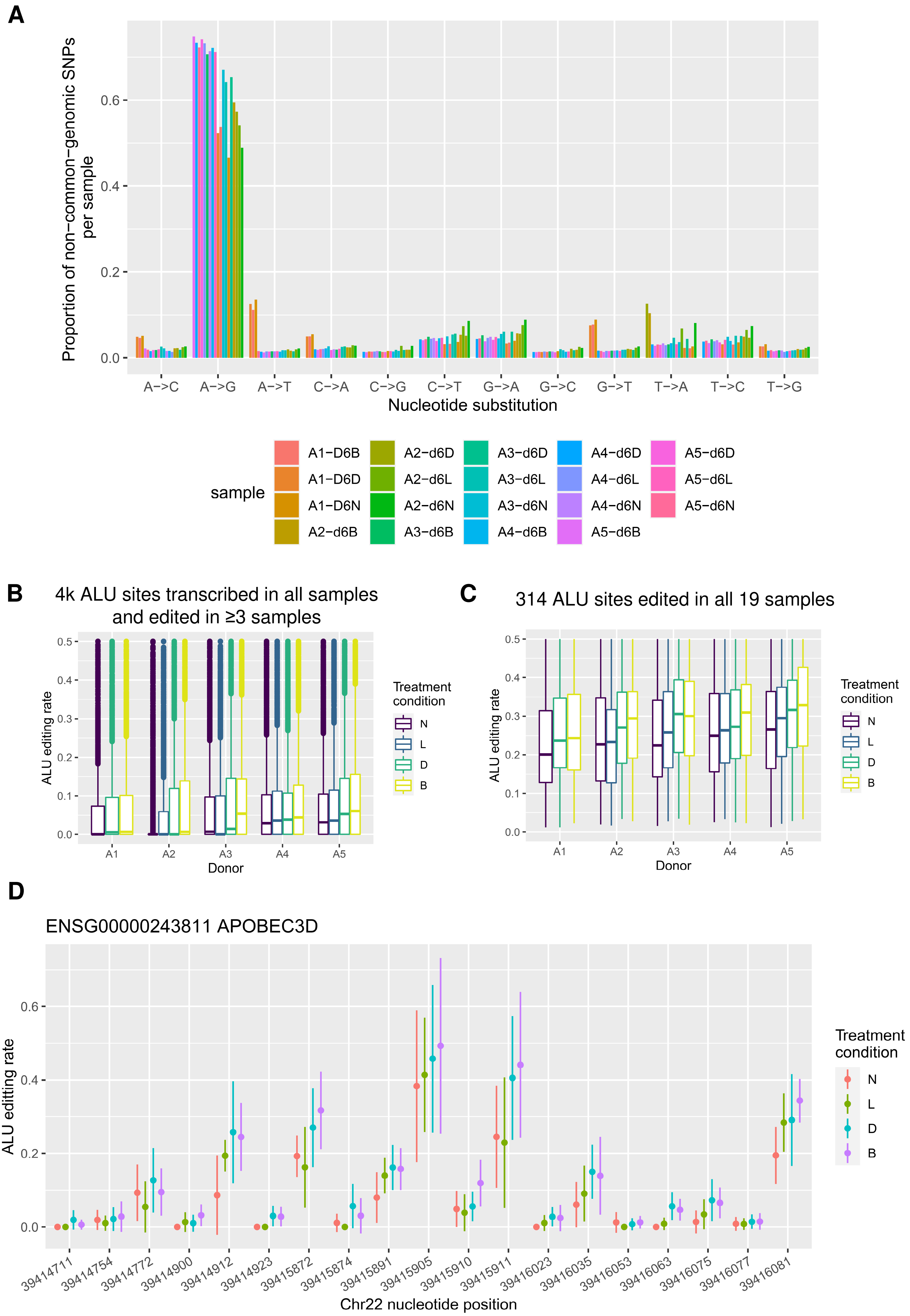
A
Proportion of non−common−genomic SNPs per sample
0.0
0.2
0.4
0.6
A−>C
A−>G
A−>T
C−>A
C−>G
C−>T
G−>A
G−>C
G−>T
T−>A
T−>C
T−>G
Nucleotide substitution
sample
A1−D6B
A1−D6D
A1−D6N
A2−d6B
A2−d6D
A2−d6L
A2−d6N
A3−d6B
A3−d6D
A3−d6L
A3−d6N
A4−d6B
A4−d6D
A4−d6L
A4−d6N
A5−d6B
A5−d6D
A5−d6L
A5−d6N
B
4k ALU sites transcribed in all samples and edited in ≥3 samples
ALU editing rate
Donor
A1
A2
A3
A4
A5
Treatment condition
N
L
D
B
C
314 ALU sites edited in all 19 samples
ALU editing rate
Donor
D
ENSG00000243811 APOBEC3D
ALU editing rate
Chr22 nucleotide position
39414711
39414754
39414772
39414900
39414912
39414923
39415872
39415874
39415891
39415905
39415910
39415911
39416023
39416035
39416053
39416063
39416075
39416077
39416081

Figure S9. Related to Figure 9. **RNA editing in BCG exposed macrophages *in vitro*.** **A**. Bar plot showing that at non-common SNPs, there is an increase in A>G editing events compared to other combinations in all RNA-seq samples used for analysis. **B**. Boxplot showing proportion of A>G per donor at 4,000 Alu sites that are transcribed in all samples and edited in at least 3 samples. **C**. Boxplot showing proportion of A>G per donor at 314 Alu sites that are transcribed and edited in all samples. **D**. A>G editing events at specific loci within APOBEC3D.

Supplementary Tables

- **Supplementary Table 1** Cytokine release in response to BCG and LPS restimulation in vitro
- **Supplementary Table 2** Gene expression difference between monocytes exposed to BCG Denmark and BCG Bulgaria
- **Supplementary Table 3** List of all dynamic genes in the in vitro BCG trained immunity model
- **Supplementary Table 4** Gene ontology terms and statistics for Figure 2C, 4D and 6C
- **Supplementary Table 5** List of genes induced by LPS restimulation in Naïve-Mf and BCG-Mf at day 6
- **Supplementary Table 6** List of MIS BAIR BCG-vaccination associated differentially methylated probes (DMPs)
- **Supplementary Table** 7 List of cisQTLs that influence BCG trained S. aureus response in the 300BCG cohort
- **Supplementary Table 8** List of MIS BAIR BCG-vaccination associated differentially methylated regions (DMRs)
